# Integrator enforces the fidelity of transcriptional termination at protein-coding genes

**DOI:** 10.1126/sciadv.abe3393

**Published:** 2021-11-03

**Authors:** Lucas Ferreira Dasilva, Ezra Blumenthal, Felipe Beckedorff, Pradeep Reddy Cingaram, Helena Gomes Dos Santos, Raghu Ram Edupuganti, Anda Zhang, Sadat Dokaneheifard, Yuki Aoi, Jingyin Yue, Nina Kirstein, Mina Masoumeh Tayari, Ali Shilatifard, Ramin Shiekhattar

**Affiliations:** 1 Department of Human Genetics, University of Miami Miller School of Medicine, Sylvester Comprehensive Cancer Center, 1501 NW 10th Avenue, Miami, FL 33136, USA.; 2 Medical Scientist Training Program and Graduate Program in Cancer Biology, University of Miami Miller School of Medicine, Miami, FL 33136, USA.; 3 Department of Biochemistry and Molecular Genetics, Feinberg School of Medicine, Northwestern University, Chicago, IL 60611, USA.; 4 Robert H. Lurie Comprehensive Cancer Center, Feinberg School of Medicine, Northwestern University, Chicago, IL 60611, USA.

## Abstract

Integrator regulates the 3′-end processing and termination of multiple classes of noncoding RNAs. Depletion of INTS11, the catalytic subunit of Integrator, or ectopic expression of its catalytic dead enzyme impairs the 3′-end processing and termination of a set of protein-coding transcripts termed Integrator-regulated termination (IRT) genes. This defect is manifested by increased RNA polymerase II (RNAPII) readthrough and occupancy of serine-2 phosphorylated RNAPII, de novo trimethylation of lysine-36 on histone H3, and a compensatory elevation of the cleavage and polyadenylation (CPA) complex beyond the canonical polyadenylation sites. 3′ RNA sequencing reveals that proximal polyadenylation site usage relies on the endonuclease activity of INTS11. The DNA sequence encompassing the transcription end sites of IRT genes features downstream polyadenylation motifs and an enrichment of GC content that permits the formation of secondary structures within the 3′UTR. Together, this study identifies a subset of protein-coding transcripts whose 3′ end processing requires the Integrator complex.

## Teaser

Integrator directly binds and terminates protein-coding transcripts enriched in alternative polyadenylation sequences.

## INTRODUCTION

Transcription by RNA polymerase II (RNAPII) occurs in three interconnected stages: initiation, elongation, and termination (*1*–*4*). While transcriptional initiation and elongation have been extensively studied, the precise steps and the protein complexes involved in the termination of transcription require further clarification (*5*). The current model of transcriptional termination in mammalian cells stipulates that as elongating RNAPII approaches the 3′ end of the gene, the heptapeptide-containing C-terminal domain (CTD) of RPB1 exhibits reduced phosphorylation of serine-5 and enhanced phosphorylation of serine-2 (*6*). These modifications coincide with the eviction of transcriptional elongation factors and recruitment of polyadenylation/termination factors, including cleavage and polyadenylation specificity factors (CPSFs) and cleavage stimulation factors (CSTFs) (*7*–*9*). The association of these factors leads to the stalling of RNAPII after it passes the polyadenylation signal (PAS) (*10*). RNA transcripts are then cleaved by the CPSF73 endonuclease 20 to 30 nucleotides (nt) downstream of the PAS (*11*). The 5′ capped and cotranscriptionally spliced mRNA is stabilized by the addition of a poly(A) tail on the 3′ end and exported to the cytoplasm for translation. The nascent transcript that remains associated with RNAPII is degraded from its exposed 5′ end by the Xrn2 exoribonuclease (*12*, *13*). Last, RNAPII is released from the DNA template most likely as a consequence of two events: an intrinsic conformational change of RNAPII triggered by arriving at the 3′ end of mRNA and the torpedo effect of Xrn2 catching up with RNAPII. Whether or not both events are required on the same transcript is debatable (*2*, *14*, *15*). Despite the extensive regulation of these multistep events, recent reports have suggested that perturbations like cellular stress or viral infection may lead to the dysregulation of termination, resulting in widespread transcriptional readthrough (*16*–*20*). Hence, the molecular alterations following these insults remain to be understood. It is also unclear whether the resulting readthrough of transcripts poses an adverse or protective effect in cellular function (*17*, *21*).

The metazoan-specific Integrator complex associates with the CTD of RNAPII and is required for 3′ end processing of numerous transcripts in the noncoding transcriptome (*22*, *23*). Integrator subunit 11 (INTS11) has endonuclease activity and forms a core module with two other subunits, INTS9 and INTS4, to cleave the 3′ end of small nuclear RNA (snRNA) involved in spliceosome formation and herpes virus primary microRNA for their proper maturation (*22*, *24*, *25*). Enhancer RNA (eRNA) is also processed in the 3′ end by Integrator, resulting in the stabilization of enhancer-promoter contacts (*23*). Previous work demonstrates that Integrator mediates the activation of immediate early genes, thereby controlling the transcriptional output of growth factor signaling (*22*, *26*, *27*). Notably, recent studies have shown that the catalytic activity of Integrator regulates elongation at coding genes by premature termination of transcripts associated with paused RNAPII (*28*–*31*).

Integrator associates with both the hypo- and hyperphosphorylated CTD (*32*). Early studies showed that phosphorylated serine-2 and serine-7 of the CTD were required for binding to Integrator in vitro (*33*). More recently, phosphorylation of tyrosine-1 on the CTD was shown to be essential for the engagement of Integrator with RNAPII in vivo. Cells with mutant tyrosine-1 displayed global defects in mRNA termination. These results suggest that the disruption of Integrator from phosphorylated tyrosine residues may partially underlie this phenotype (*34*). In addition, proteomic analyses of complexes associated with 3′ end of mRNAs revealed the presence of INTS2, INTS3, and INTS4 subunits of Integrator, providing further evidence for Integrator’s role in 3′ end processing of mRNAs (*35*). Last, depletion of INTS9 or INTS3 resulted in moderate termination defects at selected mRNAs (*36*). However, the precise role for Integrator in transcriptional termination remains to be elucidated. Considering the close sequence and structural homology of INTS11 to CPSF73, it is likely that Integrator may contribute to 3′ end cleavage of specific classes of protein-coding genes (*37*). Recent work has shown that hyperosmotic stress impairs transcriptional termination at a subset of genes by decreasing the association of Integrator with RNAPII (*38*). Furthermore, Integrator may also facilitate RNAPII pausing at termination sites through its interaction with RNAPII CTD or through the recruitment of other essential termination factors.

To investigate the role of Integrator in transcriptional termination, we performed genome-wide analysis of 3′ end processing following depletion of INTS11, the catalytically active subunit of the complex. By precisely mapping transcriptionally engaged RNAPII, we uncovered extensive defects in transcriptional termination at over 1300 genes. INTS11 binds and cleaves these pre-mRNAs at the proximal poly(A) sites, and its depletion induces extension of the 3′ untranslated regions (3′UTRs), spurious cleavages, and heterogeneous polyadenylation of transcripts. Together, these data highlight an important role for Integrator in the fidelity of transcriptional termination of protein-coding genes.

## RESULTS

### INTS11 depletion impairs transcriptional termination at protein-coding genes

**Fig. 1.**
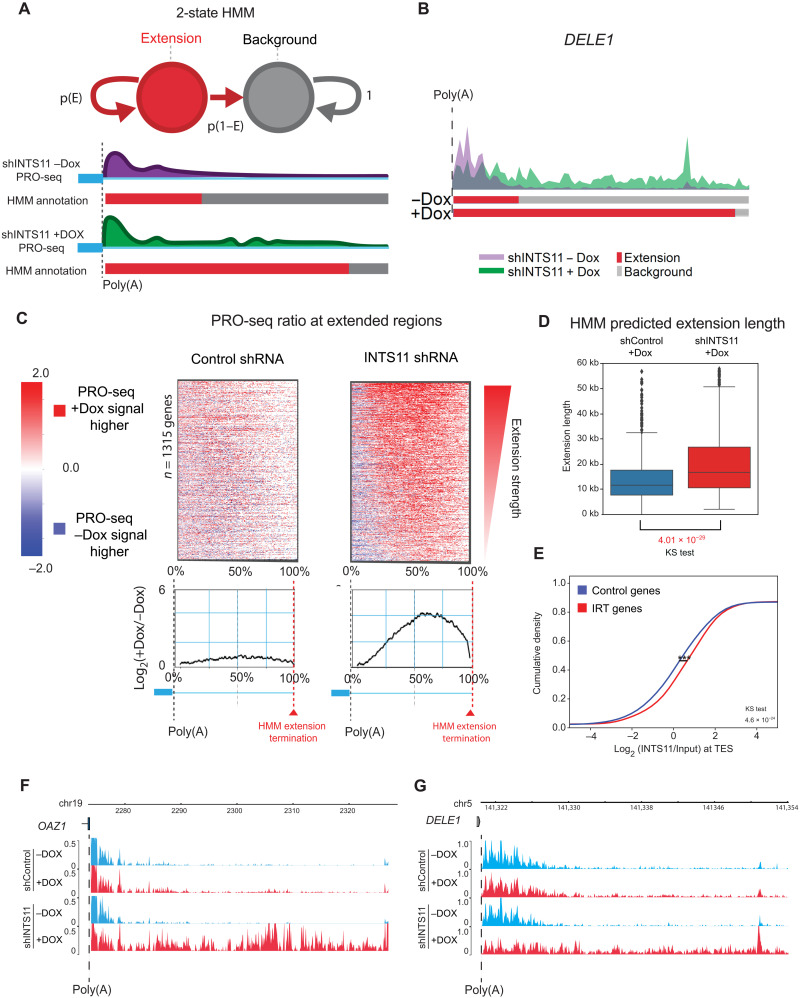
Integrator regulates transcriptional termination at protein-coding genes. (**A**) HMM algorithm used to analyze PRO-seq data at the 3′ end of genes and define IRT genes. (**B**) Example of the IRT gene DELE1 with the HMM predicted extension below. (**C**) PRO-seq heatmaps and mean density ratio spanning the poly(A) site to the HMM extension termination of 1315 IRT gene transcripts in control and INTS11-depleted cells. (**D**) Boxplot of the HMM predicted extension length of 1315 IRT gene transcripts in control and INTS11-depleted cells. (**E**) Cumulative distribution of eCLIP signal ratio log_2_(INTS11/INPUT) at TES (−/+100) of control (blue) and extended genes (red). The significance between the distribution was calculated using the Kolmogorov-Smirnov (KS) test. The control genes (*n* = 1315) used for this analysis have similar expression levels of those observed in the extended genes. (**F**) PRO-seq Genome Browser example of the IRT gene *OAZ1* at the 3′ end in control and INTS11-depleted cells. (**G**) PRO-seq Genome Browser example of the IRT gene *DELE1* at the 3′ end in control and INTS11-depleted cells.

To determine the role of Integrator in transcriptional termination at protein-coding genes, we depleted INTS11 and performed precision run-on and sequencing (PRO-seq), which assesses strand-specific nascent transcription at single-nucleotide resolution. We used a machine learning algorithm based on a two-state hidden Markov model (HMM) to analyze the PRO-seq signal at the 3′ end of protein-coding genes (see Materials and Methods and Fig. 1A). All genome-wide experiments were performed in at least two biological replicates. A number of considerations were used to identify genes whose 3′ end displayed readthrough following INTS11 depletion. We first assessed the longest isoform of protein-coding transcripts annotated by the ENSEMBL genome assembly. We next analyzed the area downstream of the CPA (cleavage and polyadenylation) site extending up to the closest expressed gene on the same DNA strand having reads per kilobase of transcripts per million mapped reads (RPKM) of >0.7 as determined by RNA sequencing (RNA-seq). HMM was then used to distinguish extended transcripts using PRO-seq reads from control and INTS11-depleted cells (Fig. 1, A and B, and fig. S1, A and B; see Materials and Methods for detailed description of HMM). This methodology revealed 1315 genes that displayed elevated PRO-seq signal downstream of canonical cleavage and poly(A) sites, which we termed Integrator-regulated termination (IRT) genes (Fig. 1, C and D, and table S1). A heatmap of IRT genes revealed spreading of PRO-seq reads beyond the poly(A) sites following INTS11 depletion (Fig. 1C). While the median HMM predicted extension length of IRT gene transcripts in control samples was around 11.6 kb, the median extension length in INTS11 knockdown conditions was 16.7 kb (Fig. 1D).

Levels of the components of the CPA complex—CSTF50, CSTF64, and CPSF100—were largely unchanged upon the loss of INTS11 (fig. S1C). We noted a 34% reduction in whole-cell levels of CPSF73; however, no change was observed in the chromatin compartment (fig. S1, D and E).

We next performed enhanced cross-linking and immunoprecipitation (eCLIP) using INTS11-specific antibodies to confirm the direct binding of INTS11 to IRT gene transcripts (*39*). There was a significant enrichment of INTS11 binding around the canonical PAS of IRT gene transcripts compared to that of control genes (Fig. 1E and fig. S1, F and G). PRO-seq Genome Browser tracks of the IRT genes *OAZ1* and *DELE1* exemplify the lengthening of transcripts upon loss of INTS11 (Fig. 1, F and G). These findings were confirmed by quantitative polymerase chain reaction (qPCR) using primers that encompass the cleavage sites of the canonical transcript and its extended downstream variant (fig. S1H).

### Increased RNAPII occupancy beyond canonical poly(A) sites following INTS11 depletion

**Fig. 2.**
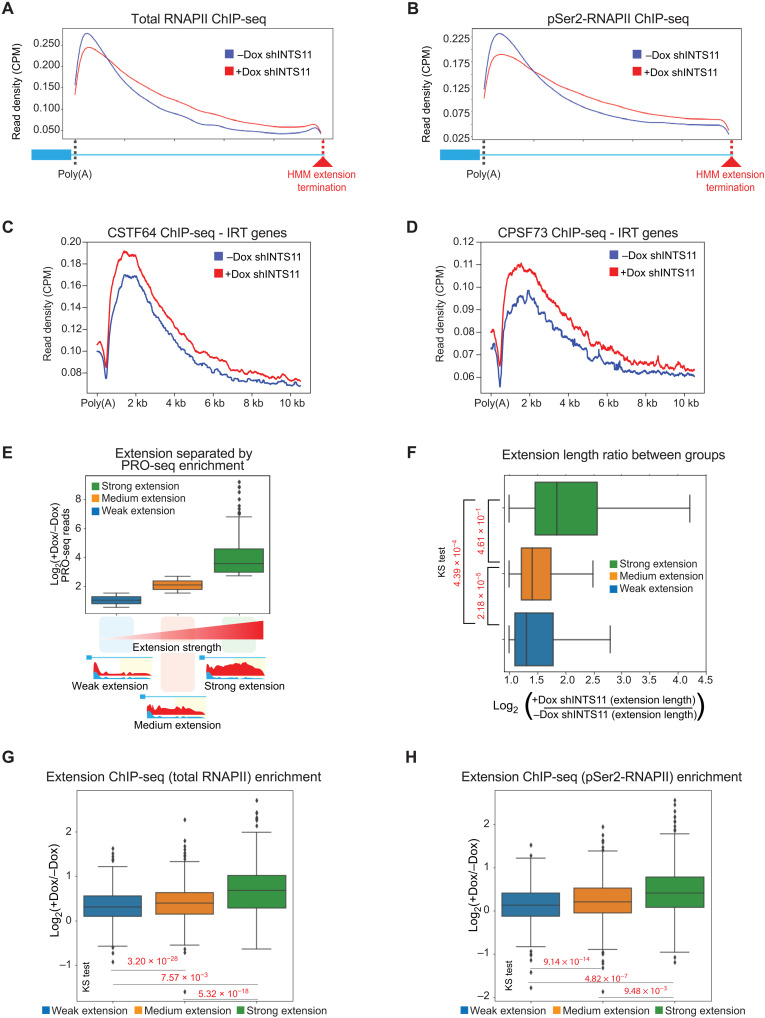
Integrator depletion induces transcriptional readthrough at protein-coding genes. (**A**) Average profile of RNAPII spanning the poly(A) site to the HMM extension termination of 1315 IRT genes in INTS11-depleted cells. (**B**) Average profile of pSer^2^-RNAPII spanning the poly(A) site to the HMM extension termination of 1315 IRT genes in INTS11-depleted cells. (**C**) Average profile of CSTF64 spanning the poly(A) site plus additional 10-kb downstream from *N*- TES. Profile at IRT genes without and with INTS11 shRNA induction. (**D**) Average profile of CPSF73 spanning the poly(A) site plus additional 10-kb downstream from TES. Profile at IRT genes without and with INTS11 shRNA induction. (**E**) Boxplot of IRT gene groups separated by PRO-seq extension strength. (**F**) Boxplot of the extension length ratio in IRT gene groups separated by PRO-seq extension strength. (**G**) Boxplot of the RNAPII extension strength in IRT gene groups separated by PRO-seq extension strength. (**H**) Boxplot of the pSer^2^-RNAPII extension strength in IRT gene groups separated by PRO-seq extension strength.

We next validated the PRO-seq results by performing chromatin immunoprecipitation and sequencing (ChIP-seq) using antibodies against total RNAPII or the elongating serine-2 phosphorylated form of RNAPII (pSer^2^-RNAPII). The occupancy of RNAPII was consistent with PRO-seq results and confirmed the presence of transcriptionally active RNAPII downstream of the canonical poly(A) site at IRT genes (Fig. 2, A and B). Our previous work demonstrates the requirement of INTS11 catalytic activity for transcriptional elongation (*28*). In line with previous findings, following INTS11 depletion, the occupancy of total RNAPII and pSer^2^-RNAPII in the gene bodies of IRT genes was reduced (fig. S2, A and B) (*28*). However, the magnitude of RNAPII reduction was greatest over the poly(A) sites of IRT genes (Fig. 2, A and B, and fig. S2, A and B). Despite the reduction of RNAPII at the 3′ end of IRT genes, ChIP-seq profiles of CSTF64 and CPSF73 in this region displayed increased occupancy after INTS11 depletion, suggesting a compensatory recruitment of the CPA complex in response to impaired termination (Fig. 2, C and D, and fig. S2, C and D).

To better understand the defects in termination incurred by the loss of Integrator, we divided the IRT genes into three classes based on the strength of 3′ end extension for each IRT transcript determined by PRO-seq (Fig. 2E and table S2). We defined the strength of extensions as the read density fold change in INTS11-depleted versus control conditions over the HMM-defined extended region. Notably, the strength of these extensions highly correlated with the length of extensions, indicating that the transcripts exhibiting the strongest extensions also displayed the longest extensions (Fig. 2F). Last, ChIP-seq of total RNAPII or the serine-2 phosphorylated form displayed a similar trend in the extension of RNAPII beyond the canonical poly(A) site, as was shown by PRO-seq analyses (compare Fig. 2E with Fig. 2, G and H). These results indicate that the depletion of INTS11 induces termination defects at a group of protein-coding genes. The failure to terminate IRT transcripts detected in nascent transcription was also reflected in the steady-state occupancy of total and serine-2 phosphorylated RNAPII beyond poly(A) sites.

### Termination defects at IRT genes manifest in their steady-state transcripts

**Fig. 3.**
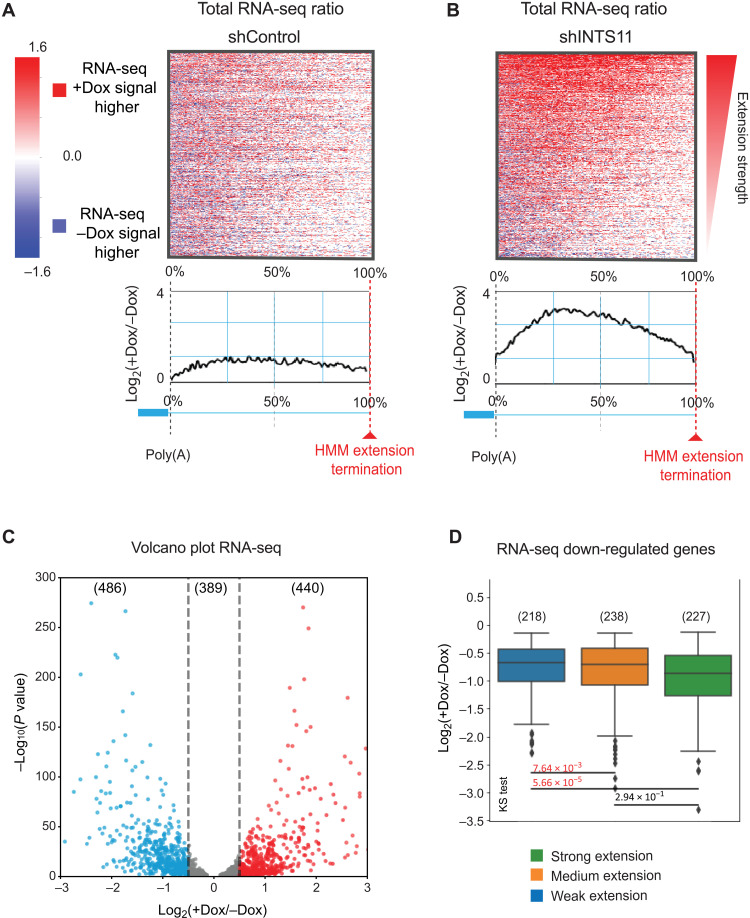
Steady-state transcripts undergo 3′ end extension following the loss of Integrator. (**A** and **B**) RNA-seq heatmaps and mean density ratio spanning the poly(A) site to the HMM extension termination of 1315 IRT gene transcripts in (A) control and (B) INTS11-depleted cells. (**C**) Volcano plot of differentially expressed IRT genes (*n* = 1315 genes, fold change = 1.5, *q* < 0.05) after INTS11 depletion. (**D**) Boxplot of down-regulated IRT genes (*q* < 0.05) separated by PRO-seq extension strength.

We performed RNA-seq to assess the consequence of 3′ end processing defects on the length and expression of steady-state mRNA produced from IRT genes. Depletion of INTS11 resulted in the 3′ end extension of IRT gene mRNA (Fig. 3, A and B), consistent with the findings observed in nascent transcripts. RNA-seq analysis revealed that more than one-third of IRT gene mRNA decreased in expression, while a similar proportion was up-regulated (Fig. 3C). While there was a lack of correlation between PRO-seq extension strength and expression levels of up-regulated IRT genes (fig. S3A), the IRT gene transcripts experiencing a greater 3′ end extension also showed greater decrease in their steady-state levels (Fig. 3D). We found the similar results using a second short hairpin RNA (shRNA) directed to a different region of INTS11 transcript (fig. S3, B to D). Together, these results confirm the PRO-seq data demonstrating IRT transcript extension beyond the canonical 3′ end following INTS11 depletion. We find that, for the IRT genes that decreased in their steady-state levels, the magnitude of mRNA reduction correlated with the degree of nascent transcript extension (Fig. 3D).

### IRT genes exhibit increased trimethylation of lysine-36 on histone H3

**Fig. 4.**
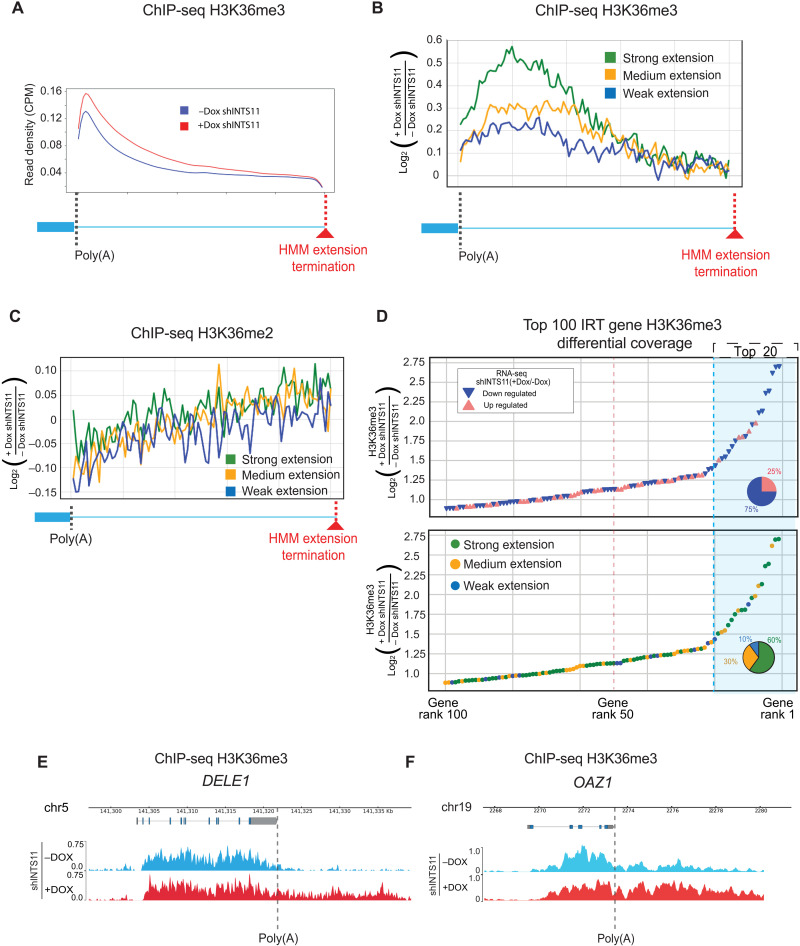
Defective termination is accompanied by de novo deposition of H3K36me3. (**A**) Average profile of H3K36me3 spanning the poly(A) site to the HMM extension termination of 1315 IRT genes in INTS11-depleted cells. (**B**) Average profile of H3K36me3 in IRT gene groups separated by PRO-seq extension strength spanning the poly(A) site to the HMM extension termination in INTS11-depleted cells. (**C**) Average profile of H3K36me2 in IRT gene groups separated by PRO-seq extension strength spanning the poly(A) site to the HMM extension termination in INTS11-depleted cells. (**D**) Mean density ratio of H3K36me3 in the extended regions of the top 100 genes ranked by differential H3K36me3 signal. The top plot denotes the differential expression status of each gene. The bottom plot displays the PRO-seq extension strength of each gene. (**E**) H3K36me3 Genome Browser example of the IRT gene *DELE1* in INTS11-depleted cells. (**F**) H3K36me3 Genome Browser example of the IRT gene *OAZ1* in INTS11-depleted cells.

Trimethylation of lysine-36 on histone H3 (H3K36me3) is an epigenetic modification that decorates the body of protein-coding genes and has been linked to exon-intron processing (*40*, *41*). Notably, we observed a robust increase in H3K36me3 beyond the poly(A) site of IRT genes following INTS11 depletion (Fig. 4A). The levels of H3K36me3 closely reflected the strength and length of extended IRT gene transcripts (Fig. 4B). In contrast, there was no change in the dimethylation of H3K36 (H3K36me2) at these genes (Fig. 4C). Notably, 75% of the top 20 genes with increased H3K36me3 in their extended regions were down-regulated in their steady-state mRNA levels. These IRT genes also displayed strong 3′ end extension in their nascent transcripts. (Fig. 4D). The de novo decoration of H3K36me3 beyond the canonical poly(A) site is exemplified by the IRT genes *DELE1* and *OAZ1* (Fig. 4, E and F). Together, we find that depletion of INTS11 induces defects in transcriptional termination at a set of protein-coding genes, resulting in transcriptional readthrough and enhanced deposition of H3K36 trimethylation.

### Dissection of IRT genes 3′ end processing reveals role for INTS11 endonuclease activity

**Fig. 5.**
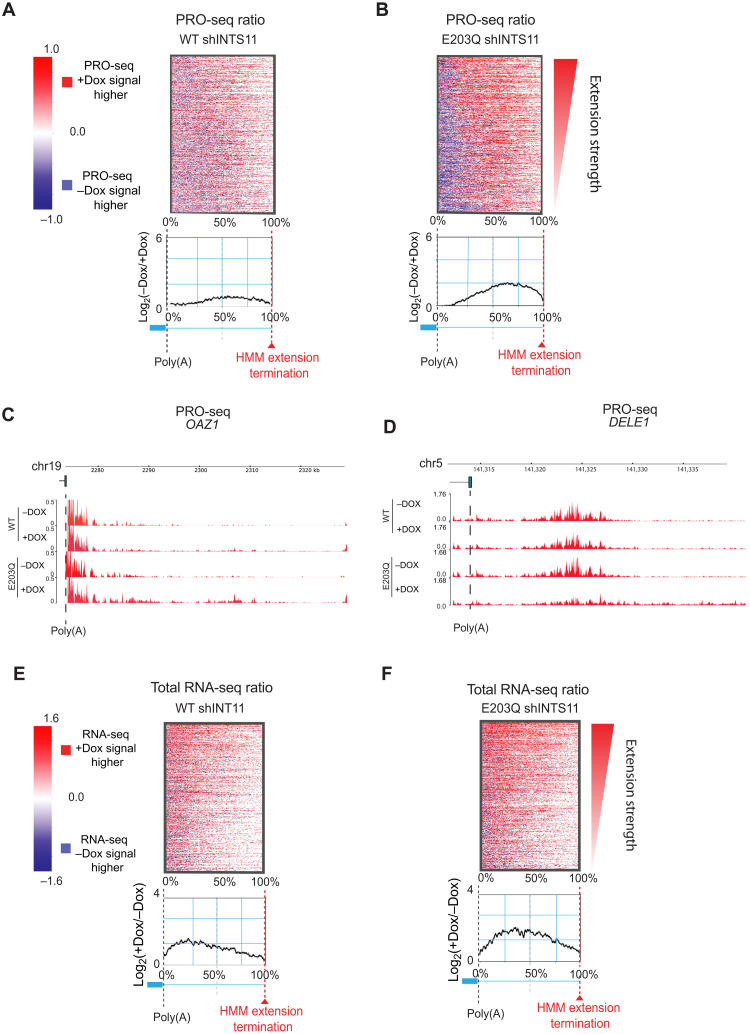
The catalytic activity of INTS11 is required for transcriptional termination at protein-coding genes. (**A** and **B**) PRO-seq heatmaps and mean density ratio spanning the poly(A) site to the HMM extension termination of 1315 IRT gene transcripts in (A) WT INTS11 and (B) E203Q INTS11 rescue cells. (**C**) PRO-seq Genome Browser example of the IRT gene *OAZ1* at the 3′ end in WT-INTS11 and E203Q-INTS11 rescue cells. (**D**) PRO-seq Genome Browser example of the IRT gene *DELE1* at the 3′ end in WT INTS11 and E203Q INTS11 rescue cells. (**E** and **F**) RNA-seq heatmaps and mean density ratio spanning the poly(A) site to the HMM extension termination of 1315 IRT gene transcripts in (E) WT INTS11 and (F) E203Q INTS11 rescue cells.

As Integrator was initially described to cleave the 3′ end of noncoding RNA (*22*–*25*), we asked whether the endonuclease activity of INTS11 is required for the 3′ end processing of IRT genes. We developed HeLa cells that stably express Flag-tagged wild-type INTS11 (WT shINTS11), or the enzymatic dead mutant INTS11 (E203Q shINTS11), in the genetic background of a doxycycline (Dox)–inducible shINTS11. WT and E203Q INTS11 proteins were expressed at similar levels from sequences refractory to the shRNA against endogenous INTS11 (fig. S4A) (*22*). A heatmap of IRT genes revealed that ectopic expression of WT INTS11 rescued termination defects incurred by INTS11 depletion, whereas expression of E203Q INTS11 sustained extensions of nascent transcripts assessed by PRO-seq (Fig. 5, A and B). Two PRO-seq examples of the IRT genes *OAZ1* and *DELE1* are shown in Fig. 5 (C and D). We next performed RNA-seq on WT and E203Q INTS11 cells to better understand the outcome of impaired INTS11 catalysis on the length of mRNA produced from IRT genes. While expression of WT INTS11 restored the fidelity of termination in steady-state transcripts, E203Q INTS11 failed to rescue the 3′ end extensions in IRT gene mRNA (Fig. 5, E and F, and fig. S4B). It is worth noting that expression of E203Q INTS11 elicited transcript misprocessing to a lesser extent than the depletion of INTS11, suggesting a possible role for other Integrator subunits in termination of IRT genes (compare Figs. 1C and 5B). Nevertheless, these results highlight the contribution of the INTS11 endonuclease activity in the 3′ end processing of IRT gene transcripts.

### Integrator contributes to proximal cleavage and prevents spurious cleavage at downstream sites of IRT genes

**Fig. 6.**
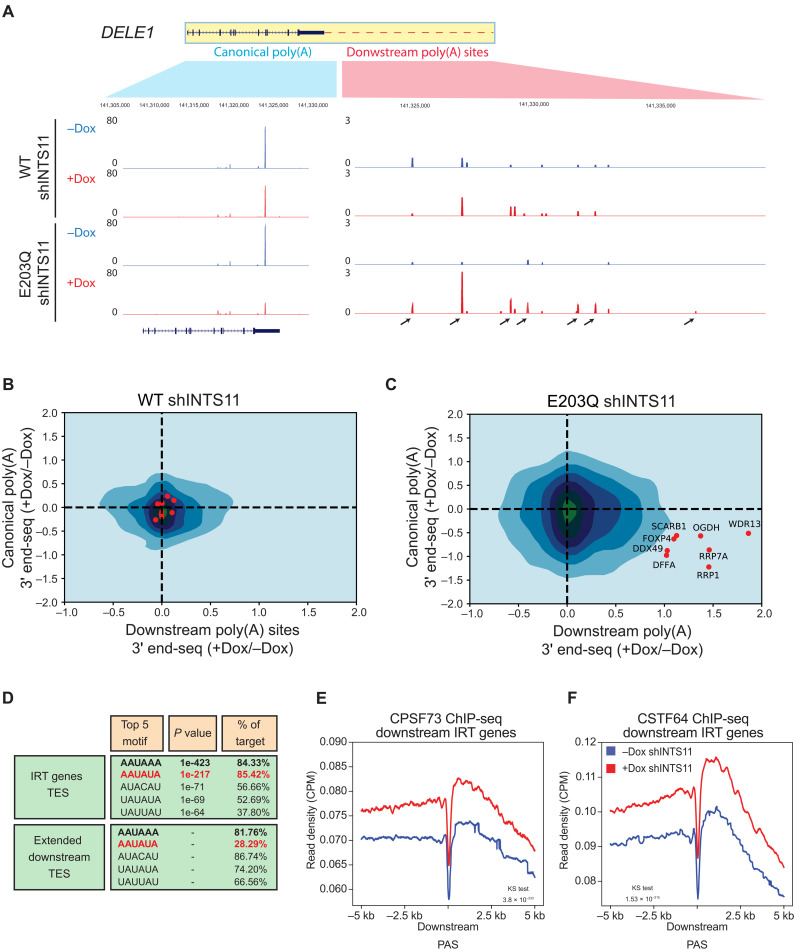
INTS11 catalyzes the 3′ end cleavage of protein-coding transcripts at canonical termination sites. (**A**) 3′ RNA-seq Genome Browser example of the IRT gene *DELE1* in WT INTS11 and E203Q INTS11 rescue cells. (**B**) CM of canonical versus downstream termination site usage in WT INTS11 rescue cells. (**C**) CM of canonical versus downstream termination site usage in E203Q INTS11 rescue cells. (**D**) Motif analysis at canonical 3′ end and downstream extended transcript 3′ end. A window of −/+150 nt from the peak was used for the analysis; top 5 motifs were shown. (**E**) CPSF73 ChIP-seq profiles at 3′ end in the downstream region of IRT genes. Profiles without (blue line) and with INTS11 shRNA induction (red line). (**F**) CSTF64 ChIP-seq profiles at 3′ end in the downstream region of IRT genes. Profiles without (blue line) and with INTS11 shRNA induction (red line).

To gain further insight into INTS11 catalysis at the 3′ end of IRT gene transcripts, we mapped the cleavage sites genome-wide using strand-specific QuantSeq 3′ mRNA-seq REV (3′ end-seq) following ectopic expression of WT or E203Q INTS11 and depletion of endogenous INTS11. This methodology uses oligo-dT during the complementary DNA (cDNA) generation to select and purify the 3′ end of polyadenylated transcripts. We found that ectopic expression of catalytic dead INTS11 in the absence of endogenous INTS11 resulted in de novo cleavages and utilization of low-frequency cleave sites downstream of the canonical site due to deficient 3′ end processing (Fig. 6A, the arrows depict these sites).

We devised a two-dimensional (2D) Gaussian distribution of these sites, which we termed the cleavage matrix (CM), to simultaneously compare alterations in canonical cleavage site usage with de novo cleavage events in extended regions of IRT gene transcripts (Fig. 6, B and C). The CM resolves processing alterations in individual transcripts at their canonical sites and de novo cut sites, which are then represented as a 2D topographic map. The color intensity in the CM displays the concentration of genes at a particular position. Ectopic expression of WT INTS11 in cells depleted of endogenous INTS11 minimally changed its CM, as most genes were centered close to the origin of the 2D graph (Fig. 6B). In contrast, ectopic expression of E203Q INTS11 in the same background induced a profound effect on cleavage site usage, as the CM coordinates of numerous genes were altered. Specifically, the downward-right shift of signal in the E203Q INTS11 CM illustrates decreased canonical cleavage usage and enhanced de novo cleavage events in the extended regions of IRT gene transcripts (Fig. 6C). Using a negative binomial generalized regression model, we found no difference in canonical cleavage site usage in control conditions (Coef = 0.114, *P* > 0.05). However, expression of E203Q INTS11 in the absence of endogenous INTS11 showed a significant difference in canonical cleavage site usage (Coef = 0.064, *P* < 0.05). Notably, the number of downstream cleavage sites significantly increased only in cells lacking the catalytic activity of INTS11 (fig. S5, A and B).

After defining the precise TES (transcript end sites) using 3′ RNA-seq, we examined the DNA motifs around canonical termination sites to better understand how sequence composition contributes to the behavior of termination at IRT genes. The canonical PAS AAUAAA was found in about 80% of IRT genes, which is close to the expected frequency found in the genome (Fig. 6D) (*42*, *43*). Notably, the alternative polyadenylation (APA) sequence AAUAUA was enriched in 85% of Integrator-dependent cleavage sites at IRT genes, while its expected frequency in the genome is lower than 30% (Fig. 6D) (*42*, *43*). The canonical motif was also present near downstream termination sites, which were occupied by the CPA complex subunits CSTF64 and CPSFT73 (Fig. 6, D to F). Notably, ChIP-seq profiles of these subunits showed increased occupancy after INTS11 depletion, suggesting that the CPA machinery terminates these extended transcripts (Fig. 6, E and F). CPA subunits were broadly detected downstream of canonical poly(A) sites and flanked both 5′ and 3′ ends of de novo poly(A) sites (Fig. 6, E and F).

We next asked whether IRT transcripts have the potential to form secondary structures around their termination sites. We found that the sequences in the vicinity of the IRT poly(A) sites were predicted to have a negative delta free energy, indicating that these transcripts spontaneously form secondary structures (fig. S5C). This difference was graded and most pronounced in IRT genes that displayed the strongest extensions (fig. S5C). Moreover, analysis of the genomic sequence around poly(A) sites showed a high CG skew in IRT genes, which are known to favor the formation of R loops in DNA sequences (fig. S5D) (*44*). Last, we sought to understand the functional pathways of IRT genes whose transcripts were strongly extended upon depletion of INTS11. These genes were divided into modules according to their top ontology terms found in uterine cervical tissue. Strongly extended genes are involved in critical cellular processes including nucleosome maintenance, organelle assembly, and nucleic acid metabolism (fig. S5E).

Together, we propose that Integrator catalyzes the 3′ end processing of pre-mRNA transcripts derived from 1315 genes enriched in APA motifs and CG content. The loss of INTS11 or its catalytic activity at these sites results in transcriptional readthrough and de novo deposition of H3K36me3 coincident with alterations in steady-state gene expression.

## DISCUSSION

**Fig. 7.**
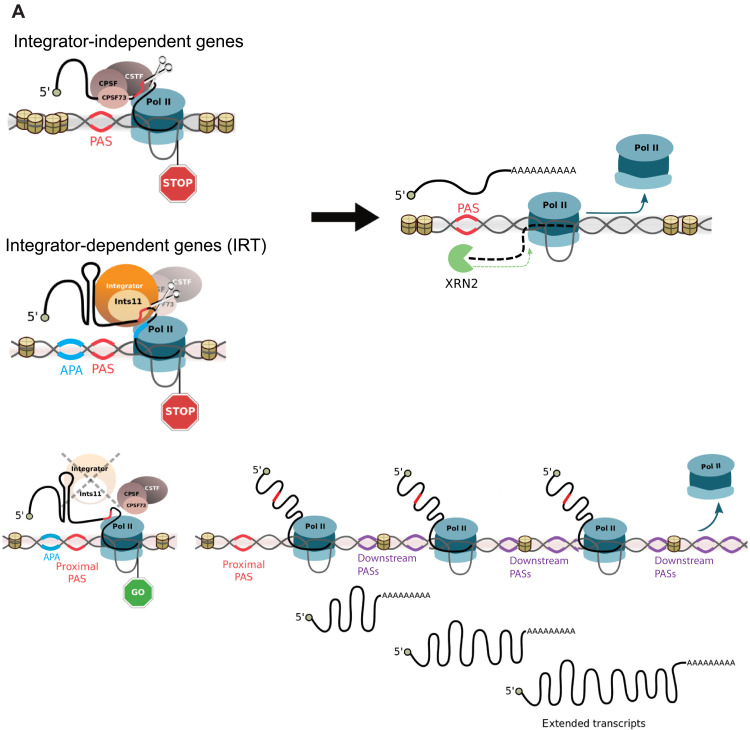
Model depicting the role of Integrator in the termination of pre-mRNA transcripts. IRT transcripts are enriched in APA sequence AAUAUA motifs. In the absence of Integrator, RNAPII moves beyond the proximal PAS sites and the extended transcripts are cleaved at multiple downstream sites.

We previously showed that Integrator is involved in the 3′ end processing of multiple classes of noncoding RNAs, including snRNAs, eRNAs, and *NEAT1* (*22*, *23*, *45*). Moreover, we recently showed that Integrator terminates transcripts associated with paused RNAPII and facilitates transcriptional elongation through the subsequent recruitment of an elongation-competent RNAPII complex (*28*). The present study reveals a role for Integrator in 3′ end processing and termination of a subset of protein-coding genes (IRT genes) that are enriched in the APA sequences. We show that the endonuclease activity of INTS11 directly contributes to the 3′ end processing and termination of transcription at IRT genes. In the absence of INTS11 cleavage activity, RNAPII transcribes beyond the canonical polyadenylation site, leading to the extension of nascent transcripts beyond their annotated 3′ end. These extensions manifest in steady-state levels of mRNA. The aberrant transcription in these areas results in an increase in H3K36me3 downstream of IRT genes and an enrichment in the CPA machinery. Therefore, at IRT genes, INTS11 endonucleolytic activity enforces 3′ end processing of transcripts with a proximal cleavage site enriched in APA sequences (Fig. 7). Our data agree with recent a publication showing that cells under hyperosmotic stress undergo transcriptional readthrough in a subset of genes that rely on Integrator to mediate the transcription termination (*38*).

The termination of transcription requires the coordination of multiple events, including cleavage at 3′ end of RNA transcripts, slowing down or pausing of elongating RNAPII, and the final dislodging of RNAPII from DNA template. Perturbations in any of these steps might have a significant impact on termination. It is likely that beyond INTS11 catalytic activity, Integrator is further required for recruitment of additional critical factors for termination. We find that depletion of INTS11 displays a greater defect in termination than the loss of its catalytic activity. The knockdown of INTS11 may destabilize the association of key subunits such as INTS9, INTS1, or INTS3 which then manifests as a cumulative impairment in 3′ end processing (fig. S1B). Moreover, it is feasible that defective processing of Integrator-dependent RNAs such as eRNAs or snRNAs could contribute to compromised termination of IRT genes. Future studies using in vitro functional reconstitution of Integrator, RNAPII, and substrate RNAs will be required to examine the detailed mechanism of transcriptional termination.

A recent study demonstrates that concomitant knockdown of INTS1 and CPSF73 induces readthrough at some sno/snRNAs, indicating that auxiliary cleavage activity may be relevant when Integrator is absent (*46*). However, our data show that the protein levels of CPSF73 and CSTF64 and their occupancy at IRT genes were unaffected by INTS11 depletion. Hence, loss of IRT 3′ end processing is not likely due to decreased recruitment of CPA complex. Notably, we observed increased genomic occupancy of CPA components at the 3′ end of IRT genes, suggesting that, in the absence of INTS11, recruitment of the CPA machinery may compensate for the lack of 3′ end processing and sustains the recruitment of subsequent termination factors. A recent study suggests that Integrator and CPA machinery can occupy similar regions of DNA and cooperate to terminate human lncRNA transcription (*30*). Our results point to cooperative roles for the Integrator complex and CPA machinery in the processing of IRT genes, thereby extending this model to protein-coding genes (Fig. 7).

Overall, there is relatively low frequency of APA sequences in the 3′UTR of genes in the human genome (*42*, *43*, *47*). Therefore, it is intriguing that we find an overrepresentation of the APA sequence motif AAUAUA in the vicinity of IRT termination sites. Current studies indicate that transcripts containing APA sequences are less efficiently processed than those containing a canonical PAS due to the presence of secondary structures that may hinder the binding of termination factors (*43*). We posit that APA sequences and secondary structural conformations of transcripts may require the action of Integrator at IRT genes. In agreement, we find that IRT gene transcripts display an entropically favorable formation of secondary structures in their 3′UTR (fig. S5A). Moreover, the GC skew around IRT poly(A) sites promotes the formation of R-loops in transcripts (fig. S5, B to D), which may necessitate Integrator cleavage function (*44*). Together, our results highlight the requirement for Integrator in the 3′ end processing of a class of protein-coding genes displaying additional sequence and secondary structure considerations in their 3′ end.

## MATERIALS AND METHODS

### Cell lines

HeLa cell lines stably expressing Dox-inducible shRNA hairpins, namely, shINTS11, shControl, WT shINTS11, and E203Q shINTS11, were established previously and were maintained in Dulbecco’s modified Eagle’s medium (DMEM) containing puromycin (2 μg/ml) (*28*). Rescue cell lines were cultured with G418 (200 μg/ml) added to the medium. Knockdowns were induced by addition of fresh Dox (1 mg/ml) in the culture medium every 24 hours for 3 days.

### Antibodies

The antibodies used in this study are as follows: for ChIP-seq, RPB1 NTD (Cell Signaling Technology, 14958, lot #1), RPB1 Ser^2^ (Abcam, 5095, lot #GR3225147-1), H3K36me3 (Abcam, 9050, lot #GR3257952-1), H3K36me2 (Abcam, 9049, lot #GR3236147-1), CSTF64 (Bethyl Laboratories, A301-92A, lot #A301-092A-2), and CPSF73 (Bethyl Laboratories, A301-019A, lot #A301-091A-1); for Western blot, INTS11 (Atlas Antibodies, HPA029025, lot #A107128), lamin A (Active Motif, 39961, lot #33310001), TFIIB (Cell Signaling Technology, 4169s, lot #1), CPSF100 (Bethyl Laboratories, A301-583A-M, lot #A301-583A-M-1), CSTF50 (Bethyl Laboratories, A301-250A-M, lot #A301-250A-M-3), CSTF64 (Bethyl Laboratories, A301-092A-M, lot #A301-092A-M-2), CPSF73 (Bethyl Laboratories, A301-091A-M, lot #A301-091A-M-1), and glyceraldehyde-3-phosphate dehydrogenase (GAPDH) (Abcam, ab8245, lot #GR3317834-1).

### Western blotting

Western blotting was performed using standard methods. All antibodies were used at dilutions recommended by the manufacturer. Western blots were imaged with the LI-COR Odyssey CLx Imaging System. The band intensities were measured and analyzed by Image Studio Lite software. Three independent experiments were conducted.

### Reverse transcription qPCR

Total RNA was extracted using TRIzol reagent and was deoxyribonuclease (DNase)–treated using the TURBO DNA-free Kit (Invitrogen, #AM1907). For cDNA synthesis, the RevertAid First Strand cDNA Synthesis Kit (Thermo Fisher Scientific) was used. The primers used in qPCR were given in table S2. qRT-PCRs were done with iTaq Universal SYBR Green Supermix (Bio-Rad). qPCR analysis was performed on a Bio-Rad CFX96 real-time system. Data were normalized to an internal gene control (*GPI*).

### Precision run-on and sequencing

PRO-seq experiments were performed and analyzed as described previously (*28*). Briefly, PRO-seq experiments were performed as described previously (*48*). Nuclei were isolated with a Dounce homogenizer with loose pestle. Nuclei (1 × 10^7^) were subjected to nuclear run-on (30°C, 3 min) in the presence of 25 mM Biotin-11-ATP/GTP/CTP/UTP (PerkinElmer). Total RNA was extracted and hydrolyzed in 0.2 M NaOH (on ice, 10 min). Biotinylated nascent RNAs were purified by Dynabeads M-280 streptavidin (Invitrogen). Following adaptor ligation, cDNA synthesis, and PCR amplification, 140– to 350–base pair (bp) libraries were size-selected by Pippin HT with 2% gel cassette 20B (Sage Science) and then sequenced using a NextSeq 500 system (Illumina) with single-read runs. Raw fastq data were trimmed by Cutadapt 1.14 (*49*) and Trimmomatic v0.32 (*50*), and then aligned on hg19 or dm3 genome by bowtie 1.1.2 (*51*). Strand-specific single-nucleotide ends of aligned reads were generated by BEDTools v2.28 with genomecov (*52*) as bedgraph format. Bedgraph data were normalized by the number of reads mapped to spike-in dm3 genome and then converted to bigwig data, which were used for downstream analyses.

### RNA-seq and data analysis

Total RNA was extracted using TRIzol reagent (Thermo Fisher Scientific, #15596026) according to the manufacturer’s instructions. Genomic DNA was removed by Turbo DNase treatment (Invitrogen, #AM1907). Total RNA-seq (ribo-depleted) libraries were produced using a TruSeq Stranded Total RNA Library Prep kit (Illumina, #20020596) with 500 ng of DNase-treated input RNA. Raw fastq RNA-seq data were processed with Trimmomatic v0.32 (*50*) and aligned to human genome (hg19 version) using STAR aligner v2.5.3a (*53*) with default parameters and RSEM v1.2.31 (*54*) to obtain expected gene counts against the human Ensembl (release 87). Differential expression was determined between shINTS11 −Dox or shINTS11 +Dox using DESeq2 (*55*) and R v3.2.3 with *q* < 0.05. For visualization on the UCSC Genome Browser, all tracks were CPM (count per million)–normalized against the total number of usable reads in that dataset using deepTools2 (*56*).

### ChIP-seq and data analysis

ChIP-seq experiments were performed and analyzed as described previously (*28*). Basically, 2 × 107 cells were cross-linked in 1% formaldehyde for 10 min at room temperature and quenched with 125 mM glycine for RNAPII and histone ChIP. For CTSF2 and CPSF3 ChIP, samples were cross-linked with Cross-link Gold (Diagenode, C01019027) according to the manufacturer’s instructions before formaldehyde fixation. ChIP-seq libraries were generated using a NEBNext Ultra II DNA Library Prep kit [New England Biolabs (NEB), #E7645S] with at least 10 ng of input DNA. Raw FASTQ data were processed with Trimmomatic v0.32 (*50*) to remove low-quality reads and then aligned to the human genome hg19 using STAR aligner v2.5.3a (*53*). We used deepTool2 (*56*) to generate normalized bigwig and heatmaps.

### eCLIP assay, mapping, and analysis

eCLIP was performed in duplicates as previously described in (*39*). Briefly, 2 × 107 HeLa cells were crosslinked by ultraviolet-C irradiation (254 nm, 400 mJ/cm^2^) and lysed on ice followed by sonication. The lysate was subjected to ribonuclease (RNase) I (Ambion) digest (40 U/ml) in the presence of murine RNase inhibitor (NEB) and Turbo DNase (4 U/ml) (Ambion). Four micrograms of INTS11 antibody (Sigma Prestige, HPA029025) was preincubated with Dynabeads M-280 Sheep Anti-Rabbit immunoglobulin G (IgG) (Invitrogen, 11204D) for 1 hour and added to the lysates for IP at 4°C for 16 hours. Two percent of the lysate was removed and stored as size-matched input controls. Coimmunoprecipitated RNA was dephosphorylated, followed by on-bead 3′ RNA adapter ligation using high-concentration T4 RNA Ligase I (NEB). IP efficiency was verified by immunoblotting of 20% of the IP samples. Input controls and 80% of the IPed INTS11-RNA complexes were run on a NuPAGE 4 to 12% bis-tris gel and transferred to nitrocellulose membrane, and the desired size range (INTS11 signal +75 kDa) was cut from the membrane for both input and IP samples. To extract RNA, nitrocellulose membranes were finely fragmented and treated with urea/proteinase K, followed by acid phenol-chloroform extraction and purification using RNA Clean & Concentrator column cleanup (Zymo Research). Size-matched input samples were also dephosphorylated and ligated to 3′ RNA adapter. After reverse transcription (AffinityScript reverse transcriptase, Agilent), excess oligonucleotides were removed with exonuclease (ExoSAP-IT, Affymetrix), and the remaining RNA was hydrolyzed by NaOH. A 3′ DNA linker was ligated to the cDNA, and the resulting library was PCR-amplified using Q5 Ultra II Master Mix (NEB). The library was size-selected by agarose gel electrophoresis and purified (MinElute, Qiagen). Single-end sequencing was performed to an average of 40 million reads per sample using Illumina NovaSeq 6000.

Data were processed according to (*39*) and https://github.com/YeoLab/eclip. After double adapter trimming (cutadapt v1.14), resulting reads were first mapped against the repetitive genome using STAR (v2.7.6a) and the unmapped output was aligned against the human genome (hg19). PCR duplicates were removed by umi_tools (v1.0.0), and the samples were visualized in UCSC. Significant INTS11 binding was determined as enrichment over size-matched input using CLIPper with a threshold of log_2_ fold enrichment > 2 and *P* < 0.001.

### eCLIP control genes

To compare the extended gene INTS11 TES eCLIP enrichment, we generated a control gene group presenting the same number of genes as the extended genes (*n* = 1315).

To avoid selecting random genes that contain expression level (RNA-seq −Dox) distribution incompatible with our extended genes, we used an interactive process that minimizes the distribution differences (KL divergence) between the RNA-seq FPKM (fragments per kilobase of exon per million mapped reads) expressions in the −Dox condition. The final control gene set that approximates the differences between the control and extended gene expression distribution was obtained by using the scikit-learn differential evolutionary algorithm (maxiter = 50, popsize = 100), minimizing the following equation:

$$\begin{array}{c} \text{minimize}\;(x\epsilon [0,1]])=\text{KL}(p\|q.x)\\ \text{subject}\;\text{to}\;\sum_{i}^{n}x_{i}=1315 \end{array}$$

where *p* is a vector representing the 1315 extended genes FPKM, *q* is a vector containing all expressed genes FPKM, *x* is a binary vector containing only zeros and 1315 ones (this vector presents the same length as *q*), and KL is the Kullback-Leibler divergence function.

### Annotating transcript 3′ extension using a two-state HMM

For each protein-coding gene annotated in Ensembl version 37 (hg19), we defined a putative extension region as the interval between the end of the most downstream 3′UTR annotation until the next exon annotated in the expressed gene contained in the same strand. A gene was considered expressed if the RNA-seq RPKM was higher than 0.7 in the shINTS11 control −Dox or +Dox condition.

In this work, we considered extensions as long contiguous regions immediately after the 3′UTR elements. Because the PRO-seq signal can present occupational variation around the annotated TES and are affected by the gene expression level, we focused our efforts to describe long extended regions that span at least twice the expected PRO-seq signal length (>5 kb). The expected signal length was computed using the position of the highest signal value in the putative extended regions. These positions presented a log-normalized distribution that showed that more than 50% of signals were ~2.4 kb apart from the annotated TES (fig. S6A). These values were extracted from PRO-seq signal from INTS11 −Dox control and were fitted using the Python2.7 scipy (‘1.2.1’) lognorm.fit function with default parameters.

PRO-seq produces data with a high signal-to-noise ratio (*48*), but some extensions can be hard to detect as the signals are weak outside the gene body regions. The first means to address this problem was to examine extensions only for genes containing minimal mean PRO-seq RPKM >0.01 in the first 1 kb. Eliminating genes with extensions having lower coverage improves identification of IRT genes because continuous PRO-seq signal immediately after the TES avoids including adjacent elements such as enhancers and nonannotated genes being classified as extensions.

Another common problem found when annotating long continuous regions is the presence of gaps between regions, which could be originated by mapping problems (repetitive regions, sequence composition, multiple hits) or low signal density. To address this issue, we used the 1D Gaussian kernel (SD = 150) from the astropy python package (‘2.0.9’) to smooth our signals over nucleotide gapped regions.

We created a univariate two-state HMM model to devise an annotation method that would be sensitive enough to detect extensions along variable length intervals that could present small gaps and changes in signal values.

The two states present on the model were defined as an “extension state” and “background state.” We used the normal distribution (μ, σ) to fit the “extension state” and a normal distribution (μ, σ!) to fit the “background state.” The distribution and transitions used are defined in the following table:

**HMM parameters**

**Table:** 

**State**	**Distribution**	**Mean**	**SD**
Extension	Gaussian	0.6	4
Background	Gaussian	0	0.00001
**From**		**to**	**Prob**
Initial		Extension	1
Background		Background	0.8
Background		Extension	0.1
Extension		Extension	0.8
Extension		Background	0.1
Background		End	0.1
Extension		End	0.1

Our focus was to find extensions that were changing length and intensity through different treatment conditions (shINTS11 −Dox and shINTS11 +Dox). We normalized the PRO-seq values for a given putative region by capturing the maximum value between the shINTS11 −Dox and shINTS11 +Dox treatment (Eq. 1) maximum values.

$$n_{\text{treatment}}^{\prime }=\frac{n_{\text{treatment}}}{\text{max}(\text{max}(n_{+\text{Dox}}),\text{max}(n_{‐\text{Dox}}))}$$ (1)

where *n* is a vector representing all PRO-seq positions in a given putative extension interval and condition (+Dox or −Dox) and *n′* is the final treatment normalized value.

The final HMM model was used to determine the length of 3′UTR extensions by calling the states using the Viterbi algorithm and selecting only predicted regions longer than 2 kb, and at least 60% of the extended region had nonzero PRO-seq signal coverage. The HMM model was created using the python package Pomegranate 0.8.1.0. Code is available upon request.

### Differentially extended 3′UTRs

We next found extensions affected by INTS11 depletion in these annotated regions. The first filter used to find the differentially extended region was created by selecting those extended regions where the length ratio (shINTS11 +Dox/shINTS11 −Dox control) presented values ≥ 1. To eliminate regions that could be differentially extending but not necessarily related with INTS11 depletion, we compared the INTS11 depletion–generated extensions with those in the shControl (+Dox/−Dox) treatments by calculating the Pearson correlation between the PRO-seq shINTS11 (+Dox/−Dox) and PRO-seq shControl (+Dox/−Dox) signal ratio. Those regions presenting correlation ≥ 0.4 were removed from our differential extension list to keep unique events manifested by INTS11 depletion.

### 3′ End RNA-seq (3′ Quant-seq) and data analysis

Total RNA was extracted with TRizol (Thermo Fisher Scientific) and treated with TURBO DNase for 30 min at 37°C. Ribosomal RNA (rRNA) was removed using NEBNext rRNA Depletion Kit v2 (NEB), and we used 1 μg of RNA as input in QuantSeq 3′ mRNA-Seq Library Prep Kit REV (Lexogen) to prepare 3′ end libraries. 3′ Quant-seq was performed on NEXTSeq 500 (Illumina) with paired-end 75-nt sequencing. Our 3′ Quant-seq analysis was performed by using the Lexogen QuantSeq data analysis pipeline, where the raw fastq data were processed with Trimmomatic v32 (*50*) and aligned to hg19 using STAR alignment tool (*53*) setting the following parameters:

--outFilterType BySJout --outFilterMultimapNmax 20 --alignSJoverhangMin 8 --alignSJDBoverhangMin 1 --outFilterMismatchNmax 999 --outFilterMismatchNoverLmax 0.1 --alignIntronMin 20 --alignIntronMax 1000000 --alignMatesGapMax 1000000 --outSAMattributes NH HI NM MD.

For visualization on the UCSC Genome Browser, all tracks were CPM-normalized against the total number of usable reads in that dataset using deepTools2 (*56*).

To create a poly(A) canonical peak position reference, we first opened a ±3 kb around the annotated TES and we calculated the mean 3′ seq signal using a 200-bp sliding window. The window containing the highest mean value was calculated for each of the −Dox WT and −Dox EQ conditions (two replicates each). We annotated the center of the bin that presented the highest signal in all four samples as our poly(A) canonical peak position (fig. S6B).

The downstream peak regions were annotated by merging replicate samples from the same treatment and using the find_peaks from the python scipy.signal package (v 1.2.1). To separate the downstream poly(A) sites from those sites corresponding to poly(A) sites from shorter isoform genes or from our reference canonical regions, we queried our reference poly(A) canonical site end position until the HMM predicted extension end position in a ±100-bp window.

The 2D 3′-seq density plot was generated by averaging the replicates and capturing the mean signal at the poly(A) canonical peak regions. For each sample (WT and EQ), we calculated the ratio between −Dox and +Dox condition. The *y* axis containing the 2D 3′-seq density plot was calculated by averaging the downstream peaks from a given extended region.

### Poly(A) canonical site usage

To test the significance of the effect of the integrator on the poly(A) canonical sites, we created a negative binomial generalized linear model (GLM). Applying this GLM was possible to disentangle the contribution of the Dox treatment in the poly(A) canonical sites (3′ prime-seq) for those generated by the gene expression changes after the Dox treatment (RNA-seq).

We used the following parameters to generate the GLM model

$$Y_{i}=\text{NegBin}(\mu_{i},\alpha );\;\text{log}(\mu_{i})=\beta_{0}+\beta_{1}X_{i}+\beta_{2}E_{i}$$

where *Y_i_* is estimated the mean read counts at the poly(A) canonical peak *I*, *X_i_* ∈ [0, 1] represents the treatment [+Dox or −Dox], *E_i_* is the RNA-seq log_2_ (+Dox/−Dox) for the gene associated with the poly(A) canonical peak *I*, a is the negative binomial dispersion coefficient (a = 1), and m is the mean counts at the poly(A) canonical peak *i*.

For each sample (WT and E203Q), we fitted a model using the python library stats models. We accessed the treatment *X_i_* [+Dox or −Dox] coefficient significance and *P* value for each fitted model.

### Motif analysis

De novo motif analysis was performed using Homer findMotifs v4.8.3 (*57*) with the parameters -rna and -len 6 (motif with 6 nt of length) and a window of ±150 bp in the 3′ end of the canonical PAS peak. Next, the 6-nt motifs identified in the canonical PAS analysis were used as input for Homer findMotifs with the parameters -rna -len 6 -find and a window of ±150 bp relative to the 3′ end of downstream peaks. These parameters were then used to calculate the frequency of these motifs in downstream sites. The identification of the canonical PAS 3′ end peak and downstream 3′ end peaks was described above.

### Extended region features

To compare the potential to form RNA structures, we opened a −100- and +20-bp window around the TES region and extracted the values from the predicted structure with the most negative delta-free energy by using RNAfold tool (version 2.4.14) (*58*). The GC skew was calculated using a sliding window of 200 bp with step size 10 considering that the GC skew equals (Gn − Cn)/(#Gn + Cn) as suggested (*59*).

## Notes

View/request a protocol for this paper from *Bio-protocol*.

## References

[1] T. Nagaike, C. Logan, I. Hotta, O. Rozenblatt-Rosen, M. Meyerson, J. L. Manley, Transcriptional activators enhance polyadenylation of mRNA precursors. Mol. Cell 41, 409–418 (2011). 21329879 10.1016/j.molcel.2011.01.022 PMC3060669

[2] T. S. Miki, S. H. Carl, H. Grosshans, Two distinct transcription termination modes dictated by promoters. Genes Dev. 31, 1870–1879 (2017). 29021241 10.1101/gad.301093.117 PMC5695088

[3] I. Jonkers, J. T. Lis, Getting up to speed with transcription elongation by RNA polymerase II. Nat. Rev. Mol. Cell Biol. 16, 167–177 (2015). 25693130 10.1038/nrm3953 PMC4782187

[4] M. Sansó, R. S. Levin, J. J. Lipp, V. Y.-F. Wang, A. K. Greifenberg, E. M. Quezada, A. Ali, A. Ghosh, S. Larochelle, T. M. Rana, M. Geyer, L. Tong, K. M. Shokat, R. P. Fisher, P-TEFb regulation of transcription termination factor Xrn2 revealed by a chemical genetic screen for Cdk9 substrates. Genes Dev. 30, 117–131 (2016). 26728557 10.1101/gad.269589.115 PMC4701974

[5] N. J. Proudfoot, Transcriptional termination in mammals: Stopping the RNA polymerase II juggernaut. Science 352, aad9926 (2016). 27284201 10.1126/science.aad9926 PMC5144996

[6] J. Zaborowska, S. Egloff, S. Murphy, The pol II CTD: New twists in the tail. Nat. Struct. Mol. Biol. 23, 771–777 (2016). 27605205 10.1038/nsmb.3285

[7] A. Meinhart, P. Cramer, Recognition of RNA polymerase II carboxy-terminal domain by 3′-RNA-processing factors. Nature 430, 223–226 (2004). 15241417 10.1038/nature02679

[8] J.-P. Hsin, J. L. Manley, The RNA polymerase II CTD coordinates transcription and RNA processing. Genes Dev. 26, 2119–2137 (2012). 23028141 10.1101/gad.200303.112 PMC3465734

[9] L. Davidson, L. Muniz, S. West, 3′ end formation of pre-mRNA and phosphorylation of Ser2 on the RNA polymerase II CTD are reciprocally coupled in human cells. Genes Dev. 28, 342–356 (2014). 24478330 10.1101/gad.231274.113 PMC3937513

[10] L. J. Core, J. J. Waterfall, J. T. Lis, Nascent RNA sequencing reveals widespread pausing and divergent initiation at human promoters. Science 322, 1845–1848 (2008). 19056941 10.1126/science.1162228 PMC2833333

[11] C. R. Mandel, S. Kaneko, H. Zhang, D. Gebauer, V. Vethantham, J. L. Manley, L. Tong, Polyadenylation factor CPSF-73 is the pre-mRNA 3′-end-processing endonuclease. Nature 444, 953–956 (2006). 17128255 10.1038/nature05363 PMC3866582

[12] N. J. Proudfoot, Ending the message: Poly(A) signals then and now. Genes Dev. 25, 1770–1782 (2011). 21896654 10.1101/gad.17268411 PMC3175714

[13] B. Tian, J. L. Manley, Alternative cleavage and polyadenylation: The long and short of it. Trends Biochem. Sci. 38, 312–320 (2013). 23632313 10.1016/j.tibs.2013.03.005 PMC3800139

[14] N. Fong, K. Brannan, B. Erickson, H. Kim, M. A. Cortazar, R. M. Sheridan, T. Nguyen, S. Karp, D. L. Bentley, Effects of transcription elongation rate and Xrn2 exonuclease activity on RNA polymerase II termination suggest widespread kinetic competition. Mol. Cell 60, 256–267 (2015). 26474067 10.1016/j.molcel.2015.09.026 PMC4654110

[15] H. Zhang, F. Rigo, H. G. Martinson, Poly(A) signal-dependent transcription termination occurs through a conformational change mechanism that does not require cleavage at the poly(A) site. Mol. Cell 59, 437–448 (2015). 26166703 10.1016/j.molcel.2015.06.008

[16] A. Vilborg, M. C. Passarelli, T. A. Yario, K. T. Tycowski, J. A. Steitz, Widespread inducible transcription downstream of human genes. Mol. Cell 59, 449–461 (2015). 26190259 10.1016/j.molcel.2015.06.016 PMC4530028

[17] A. R. Grosso, A. P. Leite, S. Carvalho, M. R. Matos, F. B. Martins, A. C. Vítor, J. M. P. Desterro, M. Carmo-Fonseca, S. F. de Almeida, Pervasive transcription read-through promotes aberrant expression of oncogenes and RNA chimeras in renal carcinoma. eLife 4, e09214 (2015). 26575290 10.7554/eLife.09214 PMC4744188

[18] A. J. Rutkowski, F. Erhard, A. L’Hernault, T. Bonfert, M. Schilhabel, C. Crump, P. Rosenstiel, S. Efstathiou, R. Zimmer, C. C. Friedel, L. Dölken, Widespread disruption of host transcription termination in HSV-1 infection. Nat. Commun. 6, 7126 (2015). 25989971 10.1038/ncomms8126 PMC4441252

[19] M. E. Nemeroff, S. M. L. Barabino, Y. Li, W. Keller, R. M. Krug, Influenza virus NS1 protein interacts with the cellular 30 kDa subunit of CPSF and inhibits 3′ end formation of cellular pre-mRNAs. Mol. Cell 1, 991–1000 (1998). 9651582 10.1016/s1097-2765(00)80099-4

[20] A. Vilborg, N. Sabath, Y. Wiesel, J. Nathans, F. Levy-Adam, T. A. Yario, J. A. Steitz, R. Shalgi, Comparative analysis reveals genomic features of stress-induced transcriptional readthrough. Proc. Natl. Acad. Sci. U.S.A. 114, E8362–E8371 (2017). 28928151 10.1073/pnas.1711120114 PMC5635911

[21] A. Vilborg, J. A. Steitz, Readthrough transcription: How are DoGs made and what do they do? RNA Biol. 14, 632–636 (2017). 26861889 10.1080/15476286.2016.1149680 PMC5449079

[22] D. Baillat, M. A. Hakimi, A. M. Näär, A. Shilatifard, N. Cooch, R. Shiekhattar, Integrator, a multiprotein mediator of small nuclear RNA processing, associates with the C-terminal repeat of RNA polymerase II. Cell 123, 265–276 (2005). 16239144 10.1016/j.cell.2005.08.019

[23] F. Lai, A. Gardini, A. Zhang, R. Shiekhattar, Integrator mediates the biogenesis of enhancer RNAs. Nature 525, 399–403 (2015). 26308897 10.1038/nature14906 PMC4718573

[24] T. R. Albrecht, S. P. Shevtsov, L. G. Mascibroda, N. J. Peart, I. A. Sawyer, M. Dundr, E. J. Wagner, Integrator subunit 4 is a ‘Symplekin-like’ scaffold that associates with INTS9/11 to form the Integrator cleavage module. Nucleic Acids Res. 46, 4241–4255 (2018). 29471365 10.1093/nar/gky100 PMC5934644

[25] M. Xie, W. Zhang, M. D. Shu, A. Xu, D. A. Lenis, D. DiMaio, J. A. Steitz, The host Integrator complex acts in transcription-independent maturation of herpesvirus microRNA 3′ ends. Genes Dev. 29, 1552–1564 (2015). 26220997 10.1101/gad.266973.115 PMC4526738

[26] J. Yue, F. Lai, F. Beckedorff, A. Zhang, C. Pastori, R. Shiekhattar, Integrator orchestrates RAS/ERK1/2 signaling transcriptional programs. Genes Dev. 31, 1809–1820 (2017). 28982763 10.1101/gad.301697.117 PMC5666678

[27] A. Gardini, D. Baillat, M. Cesaroni, D. Hu, J. M. Marinis, E. J. Wagner, M. A. Lazar, A. Shilatifard, R. Shiekhattar, Integrator regulates transcriptional initiation and pause release following activation. Mol. Cell 56, 128–139 (2014). 25201415 10.1016/j.molcel.2014.08.004 PMC4292851

[28] F. Beckedorff, E. Blumenthal, L. F. daSilva, Y. Aoi, P. R. Cingaram, J. Yue, A. Zhang, S. Dokaneheifard, M. G. Valencia, G. Gaidosh, A. Shilatifard, R. Shiekhattar, The human integrator complex facilitates transcriptional elongation by endonucleolytic cleavage of nascent transcripts. Cell Rep. 32, 107917 (2020). 32697989 10.1016/j.celrep.2020.107917 PMC7427568

[29] N. D. Elrod, T. Henriques, K.-L. Huang, D. C. Tatomer, J. E. Wilusz, E. J. Wagner, K. Adelman, The integrator complex attenuates promoter- proximal transcription at protein-coding genes. Mol. Cell 76, 738–752.e7 (2019). 31809743 10.1016/j.molcel.2019.10.034 PMC6952639

[30] S. Lykke-Andersen, K. Žumer, E. Š. Molska, J. O. Rouvière, G. Wu, C. Demel, B. Schwalb, M. Schmid, P. Cramer, T. H. Jensen, Integrator is a genome-wide attenuator of non-productive transcription. Mol. Cell 81, 514–529.e6 (2021). 33385327 10.1016/j.molcel.2020.12.014

[31] D. C. Tatomer, N. D. Elrod, D. Liang, M.-S. Xiao, J. Z. Jiang, M. Jonathan, K.-L. Huang, E. J. Wagner, S. Cherry, J. E. Wilusz, The Integrator complex cleaves nascent mRNAs to attenuate transcription. Genes Dev. 33, 1525–1538 (2019). 31530651 10.1101/gad.330167.119 PMC6824465

[32] C. C. Ebmeier, B. Erickson, B. L. Allen, M. A. Allen, H. Kim, N. Fong, J. R. Jacobsen, K. Liang, A. Shilatifard, R. D. Dowell, W. M. Old, D. L. Bentley, D. J. Taatjes, Human TFIIH kinase CDK7 regulates transcription-associated chromatin modifications. Cell Rep. 20, 1173–1186 (2017). 28768201 10.1016/j.celrep.2017.07.021 PMC5564226

[33] S. Egloff, S. A. Szczepaniak, M. Dienstbier, A. Taylor, S. Knight, S. Murphy, The integrator complex recognizes a new double mark on the RNA polymerase II carboxyl-terminal domain. J. Biol. Chem. 285, 20564–20569 (2010). 20457598 10.1074/jbc.M110.132530 PMC2898319

[34] N. Shah, M. A. Maqbool, Y. Yahia, A. Zine El Aabidine, C. Esnault, I. Forné, T.-M. Decker, D. Martin, R. Schüller, S. Krebs, H. Blum, A. Imhof, D. Eick, J.-C. Andrau, Tyrosine-1 of RNA polymerase II CTD controls global termination of gene transcription in mammals. Mol. Cell 69, 48–61.e6 (2018). 29304333 10.1016/j.molcel.2017.12.009

[35] Y. Shi, D. C. di Giammartino, D. Taylor, A. Sarkeshik, W. J. Rice, J. R. Yates III, J. Frank, J. L. Manley, Molecular architecture of the human pre-mRNA 3′ processing complex. Mol. Cell 33, 365–376 (2009). 19217410 10.1016/j.molcel.2008.12.028 PMC2946185

[36] J. R. Skaar, A. L. Ferris, X. Wu, A. Saraf, K. K. Khanna, L. Florens, M. P. Washburn, S. H. Hughes, M. Pagano, The integrator complex controls the termination of transcription at diverse classes of gene targets. Cell Res. 25, 288–305 (2015). 25675981 10.1038/cr.2015.19 PMC4349240

[37] D. Baillat, E. J. Wagner, Integrator: Surprisingly diverse functions in gene expression. Trends Biochem. Sci. 40, 257–264 (2015). 25882383 10.1016/j.tibs.2015.03.005 PMC4408249

[38] N. A. Rosa-Mercado, J. T. Zimmer, M. Apostolidi, J. Rinehart, M. D. Simon, J. A. Steitz, Hyperosmotic stress alters the RNA polymerase II interactome and induces readthrough transcription despite widespread transcriptional repression. Mol. Cell 81, 502–513.e4 (2021). 33400923 10.1016/j.molcel.2020.12.002 PMC7867636

[39] E. L. Van Nostrand, G. A. Pratt, A. A. Shishkin, C. Gelboin-Burkhart, M. Y. Fang, B. Sundararaman, S. M. Blue, T. B. Nguyen, C. Surka, K. Elkins, R. Stanton, F. Rigo, M. Guttman, G. W. Yeo, Robust transcriptome-wide discovery of RNA-binding protein binding sites with enhanced CLIP (eCLIP). Nat. Methods 13, 508–514 (2016). 27018577 10.1038/nmeth.3810 PMC4887338

[40] S. Kim, H. Kim, N. Fong, B. Erickson, D. L. Bentley, Pre-mRNA splicing is a determinant of histone H3K36 methylation. Proc. Natl. Acad. Sci. U.S.A. 108, 13564–13569 (2011). 21807997 10.1073/pnas.1109475108 PMC3158196

[41] P. Kolasinska-Zwierz, T. Down, I. Latorre, T. Liu, X. Shirley Liu, J. Ahringer, Differential chromatin marking of introns and expressed exons by H3K36me3. Nat. Genet. 41, 376–381 (2009). 19182803 10.1038/ng.322 PMC2648722

[42] B. Tian, J. Hu, H. Zhang, C. S. Lutz, A large-scale analysis of mRNA polyadenylation of human and mouse genes. Nucleic Acids Res. 33, 201–212 (2005). 15647503 10.1093/nar/gki158 PMC546146

[43] E. Beaudoing, S. Freier, J. R. Wyatt, J. M. Claverie, D. Gautheret, Patterns of variant polyadenylation signal usage in human genes. Genome Res. 10, 1001–1010 (2000). 10899149 10.1101/gr.10.7.1001 PMC310884

[44] P. A. Ginno, Y. W. Lim, P. L. Lott, I. Korf, F. Chedin, GC skew at the 5′ and 3′ ends of human genes links R-loop formation to epigenetic regulation and transcription termination. Genome Res. 23, 1590–1600 (2013). 23868195 10.1101/gr.158436.113 PMC3787257

[45] J. Barra, G. S. Gaidosh, E. Blumenthal, F. Beckedorff, M. M. Tayari, N. Kirstein, T. K. Karakach, T. H. Jensen, F. Impens, K. Gevaert, E. Leucci, R. Shiekhattar, J.-C. Marine, Integrator restrains paraspeckles assembly by promoting isoform switching of the lncRNA *NEAT1*. Sci. Adv. 6, eaaz9072 (2020). 32923585 10.1126/sciadv.aaz9072 PMC7455494

[46] L. Davidson, L. Francis, J. D. Eaton, S. West, Integrator-dependent and allosteric/intrinsic mechanisms ensure efficient termination of snRNA transcription. Cell Rep. 33, 108319 (2020). 33113359 10.1016/j.celrep.2020.108319 PMC7610016

[47] R. S. Laishram, Poly(A) polymerase (PAP) diversity in gene expression–star-PAP vs canonical PAP. FEBS Lett. 588, 2185–2197 (2014). 24873880 10.1016/j.febslet.2014.05.029 PMC6309179

[48] D. B. Mahat, H. Kwak, G. T. Booth, I. H. Jonkers, C. G. Danko, R. K. Patel, C. T. Waters, K. Munson, L. J. Core, J. T. Lis, Base-pair resolution genome-wide mapping of active RNA polymerases using precision nuclear run-on (PRO-seq). Nat. Protoc. 11, 1455–1476 (2016). 27442863 10.1038/nprot.2016.086 PMC5502525

[49] M. Martin, Cutadapt removes adapter sequences from high-throughput sequencing reads. EMBnet J. 17, 10–12 (2011).

[50] A. M. Bolger, M. Lohse, B. Usadel, Trimmomatic: A flexible trimmer for Illumina sequence data. Bioinformatics 30, 2114–2120 (2014). 24695404 10.1093/bioinformatics/btu170 PMC4103590

[51] B. Langmead, C. Trapnell, M. Pop, S. L. Salzberg, Ultrafast and memory-efficient alignment of short DNA sequences to the human genome. Genome Biol. 10, R25 (2009). 19261174 10.1186/gb-2009-10-3-r25 PMC2690996

[52] A. R. Quinlan, I. M. Hall, BEDTools: A flexible suite of utilities for comparing genomic features. Bioinformatics 26, 841–842 (2010). 20110278 10.1093/bioinformatics/btq033 PMC2832824

[53] A. Dobin, C. A. Davis, F. Schlesinger, J. Drenkow, C. Zaleski, S. Jha, P. Batut, M. Chaisson, T. R. Gingeras, STAR: Ultrafast universal RNA-seq aligner. Bioinformatics 29, 15–21 (2013). 23104886 10.1093/bioinformatics/bts635 PMC3530905

[54] B. Li, C. N. Dewey, RSEM: Accurate transcript quantification from RNA-Seq data with or without a reference genome. BMC Bioinformatics 12, 323 (2011). 21816040 10.1186/1471-2105-12-323 PMC3163565

[55] M. I. Love, W. Huber, S. Anders, Moderated estimation of fold change and dispersion for RNA-seq data with DESeq2. Genome Biol. 15, 550 (2014). 25516281 10.1186/s13059-014-0550-8 PMC4302049

[56] F. Ramírez, D. P. Ryan, B. Grüning, V. Bhardwaj, F. Kilpert, A. S. Richter, S. Heyne, F. Dündar, T. Manke, deepTools2: A next generation web server for deep-sequencing data analysis. Nucleic Acids Res. 44, W160–W165 (2016). 27079975 10.1093/nar/gkw257 PMC4987876

[57] S. Heinz, C. Benner, N. Spann, E. Bertolino, Y. C. Lin, P. Laslo, J. X. Cheng, C. Murre, H. Singh, C. K. Glass, Simple combinations of lineage-determining transcription factors prime cis-regulatory elements required for macrophage and B cell identities. Mol. Cell 38, 576–589 (2010). 20513432 10.1016/j.molcel.2010.05.004 PMC2898526

[58] A. R. Gruber, R. Lorenz, S. H. Bernhart, R. Neubock, I. L. Hofacker, The Vienna RNA websuite. Nucleic Acids Res. 36, W70–W74 (2008). 18424795 10.1093/nar/gkn188 PMC2447809

[59] P. Jenjaroenpun, T. Wongsurawat, S. Sutheeworapong, V. A. Kuznetsov, R-loopDB: A database for R-loop forming sequences (RLFS) and R-loops. Nucleic Acids Res. 45, D119–D127 (2017). 27899586 10.1093/nar/gkw1054 PMC5210542

