# Supplementary material for: Integrator enforces the fidelity of transcriptional termination at protein-coding genes
Source: Sci Adv. 2021 Nov 3;7(45):eabe3393. doi: 10.1126/sciadv.abe3393 (PMC8565846; doi:10.1126/sciadv.abe3393)
Supplement: Supplementary file 1 — Figs. S1 to S6 Legends for tables S1 and S2 [file sciadv.abe3393_sm.pdf]

Science Advances
AAAS

Supplementary Materials for

Integrator enforces the fidelity of transcriptional termination at protein-coding genes

Lucas Ferreira Dasilva, Ezra Blumenthal, Felipe Beckedorff, Pradeep Reddy Cingaram, Helena Gomes Dos Santos, Raghu Ram Edupuganti, Anda Zhang, Sadat Dokaneheifard, Yuki Aoi, Jingyin Yue, Nina Kirstein, Mina Masoumeh Tayari, Ali Shilatifard, Ramin Shiekhattar*

*Corresponding author. Email: rshiekhattar@med.miami.edu

Published 3 November 2021, *Sci. Adv.* **7**, eabe3393 (2021)
DOI: 10.1126/sciadv.abe3393

The PDF file includes:

Figs. S1 to S6
Legends for tables S1 and S2

Other Supplementary Material for this manuscript includes the following:

Tables S1 and S2

Supplementary Material

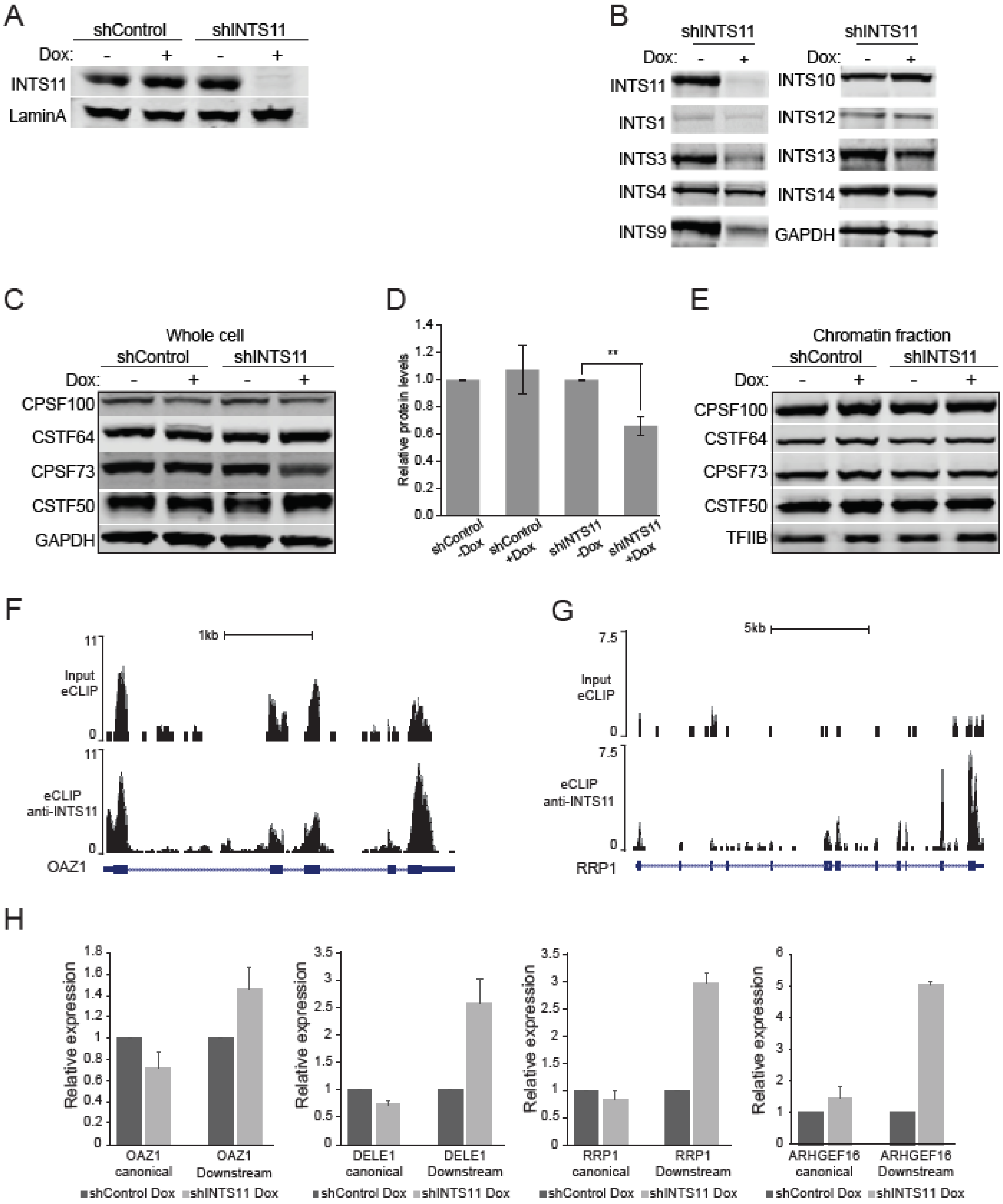

Fig. S1. Validation of INTS11 depletion and IRT gene extensions. (**A**) Western blot validation of INTS11 knockdown in shControl and shINTS11 cells. (**B**) Western blot of Integrator subunits in shINTS11 cells without (-) or with (+) the addition of doxycycline. (**C**) Whole-cell western blot of CSTF64, CSTF50, CPSF73 and CPSF100 in shControl and shINTS11 cells without (-) or with (+) the addition of doxycycline. (**D**) Western blot image quantification of CPSF73 (n=3, ** = $p<0.001$). (**E**) Chromatin fraction western blot of CSTF64, CSTF50, CPSF73 and CPSF100 in shControl and shINTS11 cells without (-) or with (+) the addition of doxycycline. (**F**-**G**) INTS11 eCLIP genome-browser example of the IRT genes (**F**) *OAZ1* and (**G**) *RRP1*. (**H**) RT-qPCR measuring the transcript levels of canonical and downstream TES of *OAZ1*, *DELE1*, *RRP1*, and *ARHGEF16* genes. The average of four technical replicates from two independent experiments is shown.

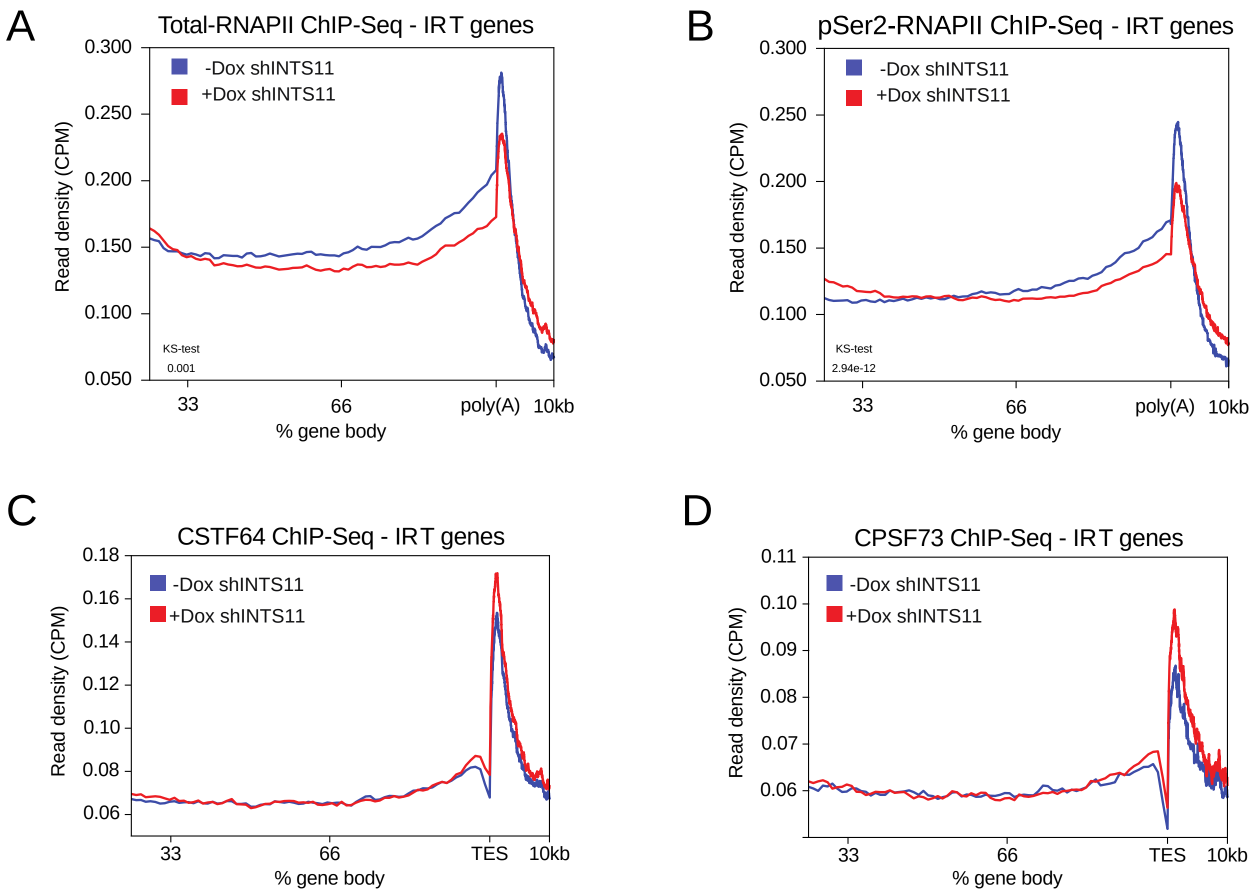

Fig. S2. RNAPII occupancy at IRT genes. (**A**) Total RNAPII ChIP-seq gene body plus additional 10kb downstream from the canonical TES. Profile at IRT genes without and with INTS11 shRNA induction. (**B**) pSer2-RNAPII ChIP-seq gene body plus additional 10kb downstream from the canonical TES. Profile at IRT genes without and with INTS11 shRNA induction. (**C**) CSTF64 ChIP-seq gene body plus additional 10kb downstream from TES. Profile at IRT genes without and with INTS11 shRNA induction. (**D**) CPSF73 ChIP-seq gene body plus additional 10kb downstream from TES. Profile at IRT genes without and with INTS11 shRNA induction.

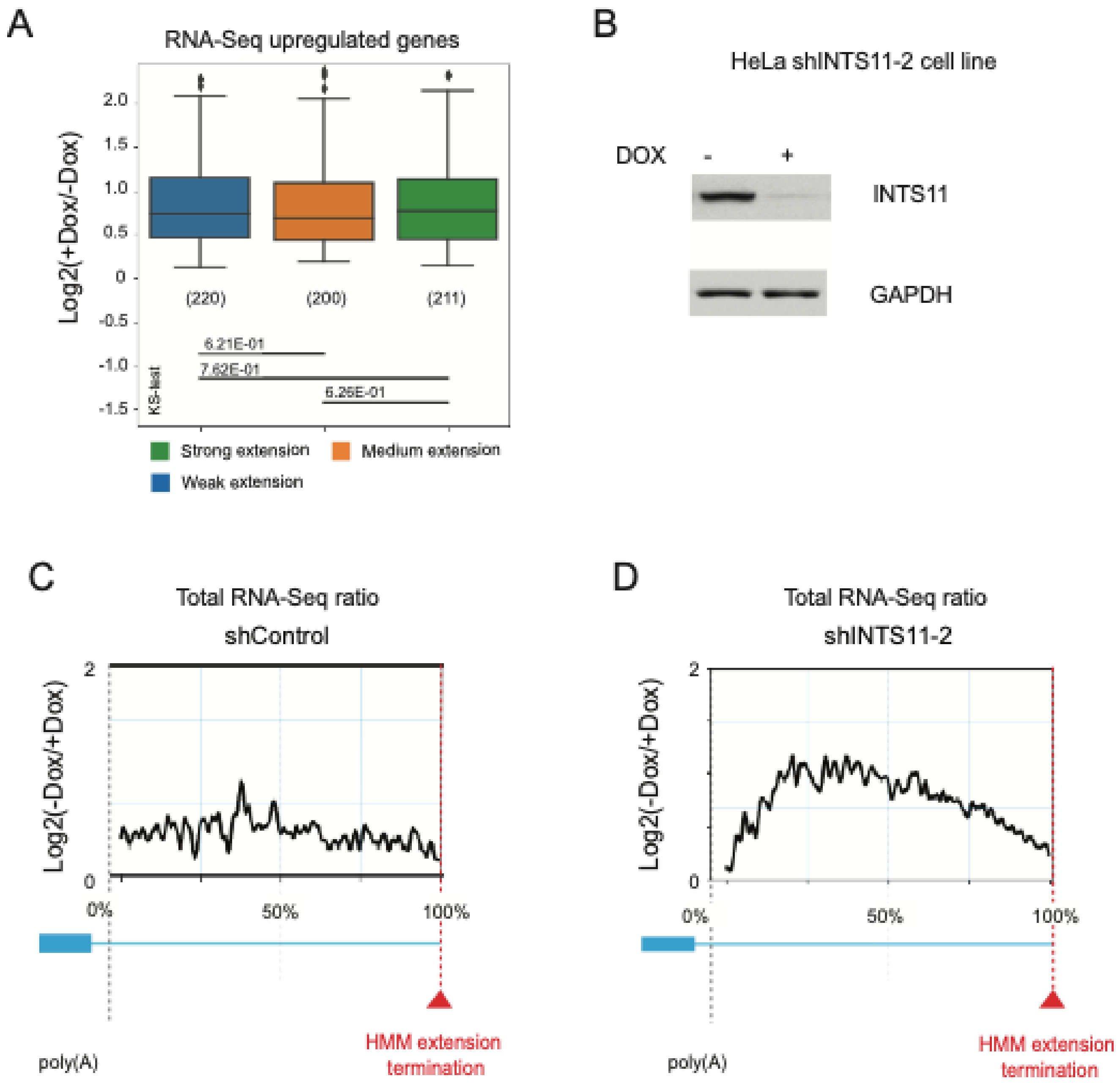

Fig. S3. Validation of IRT gene transcript extension using an alternative INTS11 shRNA. (**A**) Boxplot of upregulated IRT genes (q-value < .05) separated by PRO-seq extension strength. (**B**) Western blot validation of INTS11 depletion in shINTS11-2 cells. (**C-D**) RNA-seq mean density ratio spanning the poly(A) site to the HMM extension termination of 1315 IRT gene transcripts in (**C**) Control and (**D**) INTS11-depleted cells (shINTS11-2).

A

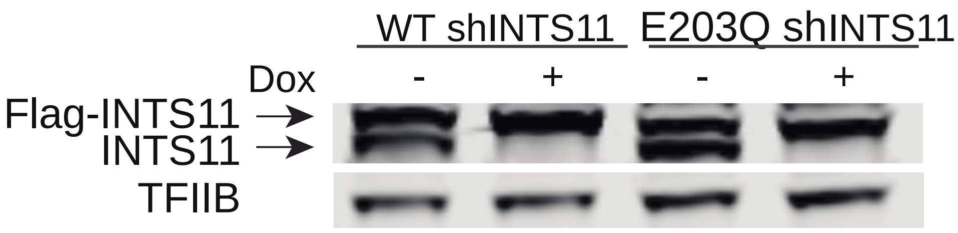

B

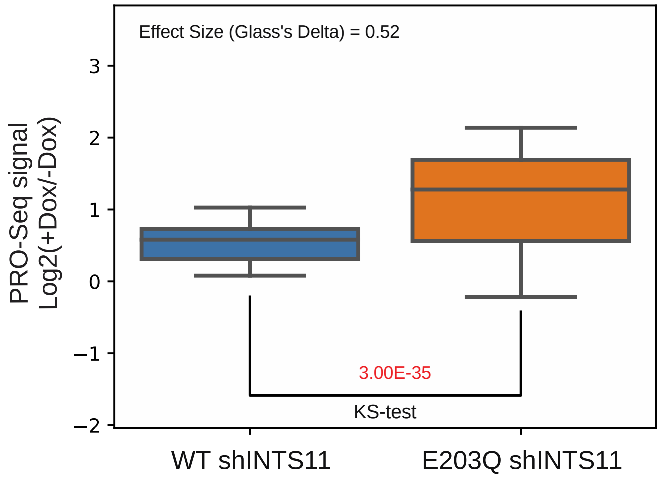

Fig. S4. Validation of INTS11 rescue cells. (**A**) Western blot validation of endogenous INTS11 knockdown and expression of WT-INTS11 and E203Q-INTS11. (**B**) Box plot showing the difference between the mean (-DOX/+DOX) fold changes at the extended regions between WT and E203Q conditions. The KS-test was used to show the distribution differences. The average effect size of WT-INTS11 vs. E203Q-INTS11 was calculated using the mean and standard deviation between samples using the Glass's Delta method.

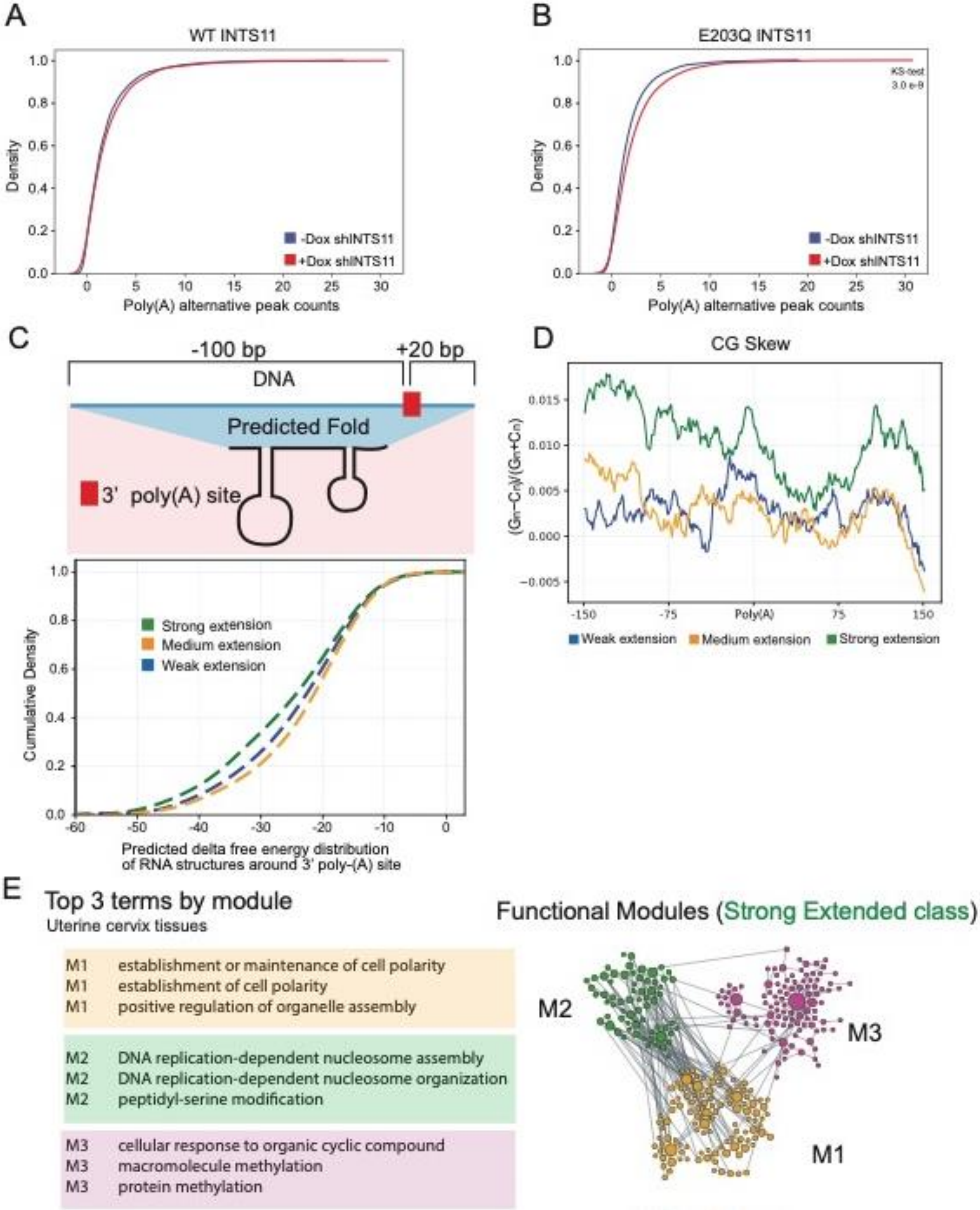
A
WT INTS11
Density
Poly(A) alternative peak counts
-Dox shINTS11
+Dox shINTS11
B
E203Q INTS11
KS-test
3.0 e-9
Density
Poly(A) alternative peak counts
-Dox shINTS11
+Dox shINTS11
C
-100 bp
+20 bp
DNA
Predicted Fold
3' poly(A) site
Strong extension
Medium extension
Weak extension
Cumulative Density
Predicted delta free energy distribution
of RNA structures around 3' poly-(A) site
D
CG Skew
Poly(A)
Weak extension
Medium extension
Strong extension
E
Top 3 terms by module
Uterine cervix tissues
M1 establishment or maintenance of cell polarity
M1 establishment of cell polarity
M1 positive regulation of organelle assembly
M2 DNA replication-dependent nucleosome assembly
M2 DNA replication-dependent nucleosome organization
M2 peptidyl-serine modification
M3 cellular response to organic cyclic compound
M3 macromolecule methylation
M3 protein methylation
Functional Modules (Strong Extended class)
M2
M3
M1

Fig. S5. Analysis of secondary structures and GC skew in the 3'UTR of IRT gene transcripts. Peak density quantification in the downstream cleavage sites in WT and E203Q in the -Dox (blue) and +Dox (red) condition. The peak count is the sum of the common signal in extended regions found in both replicates. (**A**) WT (ks-Test 0.41) and (**B**) E203Q (ks-Test 3e-09). (**C**) Free energy density plot of secondary structures around poly(A) sites in IRT gene transcripts. (**D**) GC skew of sequences around IRT gene poly(A). (**E**) Gene ontology terms of strongly extended IRT genes organized by functionality.

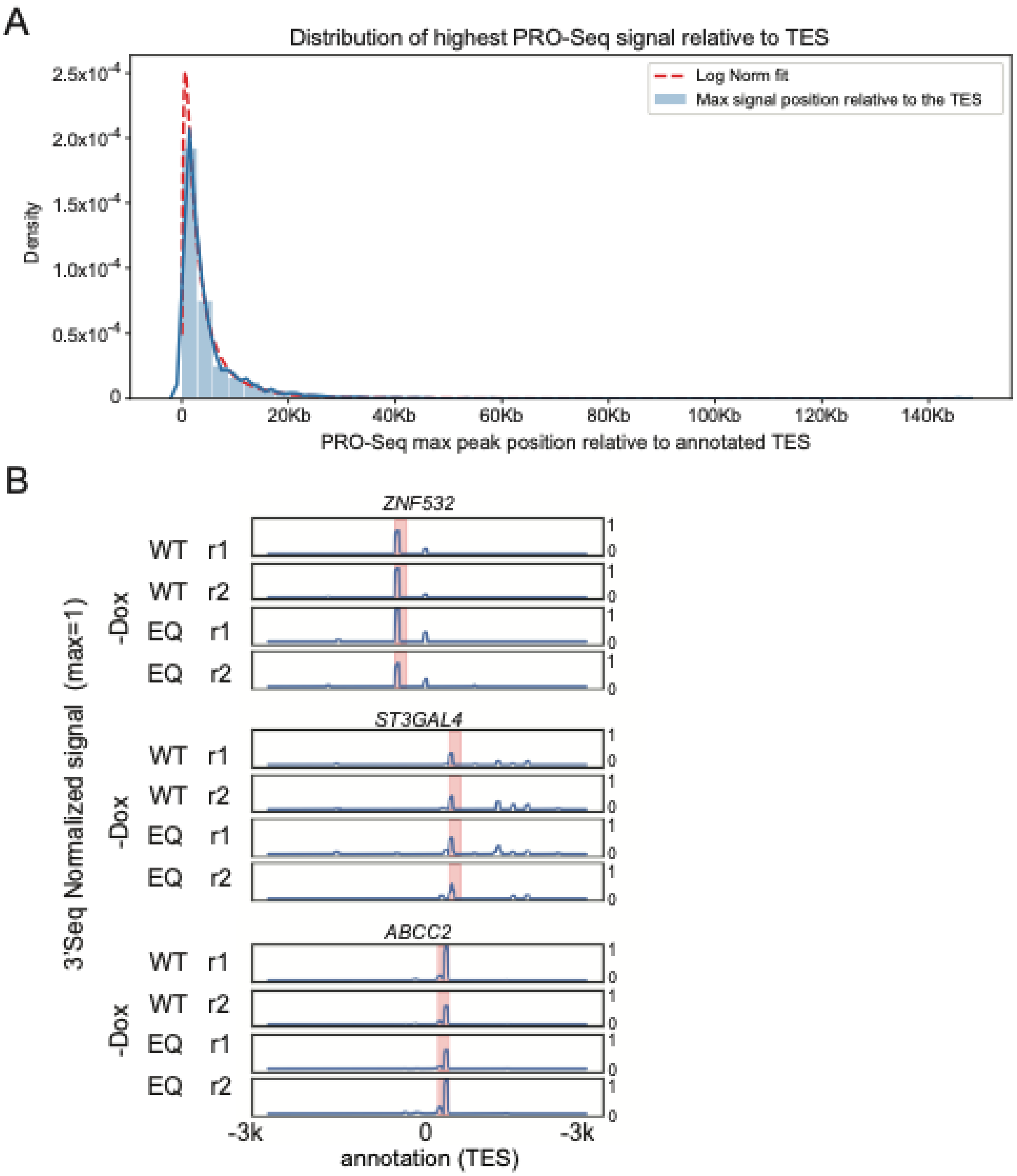

Fig. S6. Transcriptional activity around IRT TES. (**A**) Distribution of distance between annotated TES and highest PRO-Seq signal (blue) fitted to a logNormal distribution (red). (**B**) 3' Seq examples depicting strongly utilized cleavage sites.

Additional files that cannot be embedded into this Word files are the following data files (excel tables)

Table S1. IRT genes and features. Table containing several features related to the IRT genes (HMM Extension start/end position, RNA-Seq RPKM , PRO-Seq, IRT groups, canonical and downstream 3'end peaks and eCLIP control genes).

Table S2. Sequence of the RT-qPCR primers.
